# The fate of intracellular S1P regulates lipid droplet turnover and lipotoxicity in pancreatic beta-cells

**DOI:** 10.1016/j.jlr.2024.100587

**Published:** 2024-06-29

**Authors:** Yadi Tang, Mariola Majewska, Britta Leß, Ilir Mehmeti, Philipp Wollnitzke, Nina Semleit, Bodo Levkau, Julie D. Saba, Gerhild van Echten-Deckert, Ewa Gurgul-Convey

**Affiliations:** 1 Institute of Clinical Biochemistry, Hannover Medical School, Hannover, Germany; 2 Institute of Molecular Medicine III, University Hospital Düsseldorf and Heinrich Heine University, Düsseldorf, Germany; 3 Division of Hematology/Oncology, Department of Pediatrics, University of California. San Francisco, Oakland, CA, USA; 4 Life & Medical Sciences Institute, University Bonn, Bonn, Germany

**Keywords:** diabetes, beta-cells, lipid droplets, free fatty acids, insulin-secreting cells, sphingosine-1 phosphate, ceramide, mitochondria

## Abstract

Lipotoxicity has been considered the main cause of pancreatic beta-cell failure during type 2 diabetes development. Lipid droplets (LD) are believed to regulate the beta-cell sensitivity to free fatty acids (FFA), but the underlying molecular mechanisms are largely unclear. Accumulating evidence points, however, to an important role of intracellular sphingosine-1-phosphate (S1P) metabolism in lipotoxicity-mediated disturbances of beta-cell function. In the present study, we compared the effects of an increased irreversible S1P degradation (S1P-lyase, SPL overexpression) with those associated with an enhanced S1P recycling (overexpression of S1P phosphatase 1, SGPP1) on LD formation and lipotoxicity in rat INS1E beta-cells. Interestingly, although both approaches led to a reduced S1P concentration, they had opposite effects on the susceptibility to FFA. Overexpression of SGPP1 prevented FFA-mediated caspase-3 activation by a mechanism involving an enhanced lipid storage capacity and prevention of oxidative stress. In contrast, SPL overexpression limited LD biogenesis, content, and size, while accelerating lipophagy. This was associated with FFA-induced hydrogen peroxide formation, mitochondrial fragmentation, and dysfunction, as well as ER stress. These changes coincided with the upregulation of proapoptotic ceramides but were independent of lipid peroxidation rate. Also in human EndoC-βH1 beta-cells, suppression of SPL with simultaneous overexpression of SGPP1 led to a similar and even more pronounced LD phenotype as that in INS1E-SGPP1 cells. Thus, intracellular S1P turnover significantly regulates LD content and size and influences beta-cell sensitivity to FFA.

Type 2 diabetes mellitus (T2DM) is a major metabolic disorder affecting millions of people worldwide (1). In addition to hyperglycemia caused by the dysfunction of pancreatic beta-cells that secrete insulin and to insulin resistance of metabolic tissues, the disease is associated with chronic hyperlipidemia (1). Chronic exposure of pancreatic beta-cells to free fatty acids (FFA) is believed to play a crucial role in the activation of integrated stress response, eventually leading to beta-cell dysfunction and death (2, 3, 4, 5, 6). Lipotoxicity in beta-cells has been associated with *i*) oxidative stress, *ii*) endoplasmic reticulum (ER) and mitochondrial disturbances, *iii*) dysregulation of autophagy, *iv*) biosynthesis of complex lipid species [e.g., sphingolipids such as ceramide and sphingosine-1-phosphate (S1P)], and *v*) decreased lipid storage capacity (2, 3). The effects of various FFA differ substantially, depending on their chemical structure and metabolic pathways induced at various subcellular sites (5, 6, 7). Interestingly, in vitro experiments using beta-cell lines strongly indicate that monounsaturated fatty acids such as oleate (OA), which are protective in rodent beta-cells (2, 8, 9), are toxic to human beta-cells (5, 8, 10, 11); however, the underlying mechanisms remain unclear. Moreover, the sensitivity to FFA of isolated human islets is characterized by heterogeneity, similarly to a profound variability of disease progression and severity in T2DM patients.

Interestingly, recent studies indicate that S1P might be a crucial element in this context. An elevated plasma concentration of S1P has been shown to coincide with insulin resistance, obesity, and hyperinsulinemia (12, 13, 14). S1P belongs to a complex family of sphingolipids, which are biosynthesized mainly from palmitate (PA) and L-serine (condensation of palmitoyl-CoA with Ser in the ER). Additionally, use of other FFA (e.g., OA) and amino acids, though with a lower efficiency and particularly upon a PA shortage (15), has been reported resulting in the generation of atypical sphingolipids, which represent approximately 15% of sphingolipids in human plasma (16). In vitro incubations with S1P have been shown to potentiate glucose-induced insulin secretion and to stimulate beta-cell proliferation (17, 18), which was associated with elevated cAMP generation mediated by the stimulation of S1P receptors on beta-cells. The specific effects of intracellular S1P are influenced by the subcellular localization of its biosynthesis (19, 20, 21, 22). S1P can be generated from sphingosine by the action of two isoenzymes, sphingosine kinase 1 (SK1), active primarily at the plasma membrane, and sphingosine kinase 2 (SK2), localized to the ER, mitochondria, and nucleus (23). Of note, pancreatic beta-cells express predominantly SK2 (24). Intriguingly, the two SKs appear to have opposite effects on the toxicity of PA, the most abundant saturated fatty acid in human serum. Thus, SK2 knockdown was reported to protect beta-cells against PA toxicity (21, 22), while similar results were obtained in SK1 overexpressing cells (19). Once generated, S1P can undergo two fates leading to its decreased concentration: *i*) dephosphorylation (recycling) by S1P phosphatases 1 or 2 (SGPP1 or 2) back to sphingosine or *ii*) irreversible cleavage by S1P lyase (SPL) into ethanolamine phosphate and hexadecenal (25, 26). The SGPP-derived sphingosine can be re-phosphorylated to S1P or used as a substrate for ceramide generation by ceramide synthases (25). Elevated intracellular ceramide formation has been shown to mediate PA toxicity in beta-cells (19, 27, 28, 29, 30). Note that beta-cells express predominantly SGPP2 (17), which has been shown to play an important role in apoptosis in other cell types (31). In contrast, SGPP1, which is related to antiapoptotic effects in other tissues, is very weakly expressed in beta-cells (17). To our knowledge, no data regarding the role of SGPP1 in lipotoxicity in beta-cells have been published so far. The observations made in a SGPP2 KO mouse model indicate that SGPP2 is involved in beta-cell proliferation and ER function (32). The S1P-degrading enzyme SPL is expressed in a low/medium range in rodent beta-cells, while being abundantly expressed in human beta-cells (8, 17). Recently, we have shown that SPL may be crucially involved in the lipotoxic beta-cell death, an observation that coincided with a decreased number of lipid droplets (LD) in response to OA (8). Indeed, sphingolipids have been shown to regulate LD biogenesis in various cell types (33, 34), though the role of S1P turnover in this context remains unclear (33, 34).

LDs are fat storage organelles that are formed in the ER and are composed of a neutral lipid hydrophobic core and an external phospholipid monolayer containing specific proteins, such as Plin 1–5 (perilipins 1–5) (35, 36). LDs are hubs of cellular lipid and energy metabolism, and their contact with other cell organelles, such as mitochondria or lysosomes is crucial for lipid transfer and cell metabolism (35). The biogenesis of LDs continues unabated when ER function is well maintained, and it is regulated by the intracellular content of specific lipids, including neutral lipids and ceramides, particularly in the acylated form (37), by the activity of enzymatic lipid metabolism machinery (such as Dgat2) and by a proper expression of various LD-associated proteins, such as seipin or Plin 1-5 (36). The mechanism of LD turnover depends on their size and is regulated by lipophagy and lipolysis (38). LDs modulate cell fate, ER stress, and mitochondrial dysfunction by isolating lipids and inhibiting lipotoxicity (33, 34).

The role of LDs in beta-cells still remains rather unclear. In some studies, LD formation was correlated with OA-mediated protection against PA toxicity in rodent beta-cells, while others failed to observe such a link (7, 9, 39). Enhanced incorporation of PA into LDs has been recently proposed as a mechanism of the stearoyl-CoA desaturase 1 (SCD1)-mediated protection of beta-cells (40).

In the present study, we used two approaches to influence S1P metabolism: *i*) overexpression of SGPP1 and *ii*) overexpression/suppression of SPL. This strategy enabled us to discern whether a decreased concentration of S1P as such regulates the sensitivity of beta-cells to FFA or if additional aspects including the compartmentalization and activation of specific metabolic pathways in response to upregulation of each of these two enzymes are key regulatory mechanisms in LD turnover and lipotoxic beta-cell damage. We show that reduction of S1P by directing its metabolism towards recycling or cleavage indeed causes profound perturbations of LD turnover yet by different molecular mechanisms resulting in distinct sensitivity to FFA-mediated toxicity.

## Materials and Methods

### Cell culture and FFA incubations

Rat insulin-secreting INS1E cells (a kind gift of Prof. C. Wollheim, Geneva, Switzerland) were cultured in humidified atmosphere at 37°C and 5% CO_2_. The cell lines used were routinely checked for mycoplasma and were free from mycoplasma contamination. INS1E cells were cultured in RPMI 1640 medium supplemented with 10 mM glucose, 10% fetal calf serum, penicillin and streptomycin, 10 mM Hepes (Serva, Heidelberg, Germany), 2 mM glutamine, 1 mM sodium-pyruvate (Sigma-Aldrich, Taufkirchen, Germany), and 50 μM of 2-mercaptoethanol (8, 17). EndοC-βH1 beta-cells were cultured onto coated dishes or plates (fibronectin and ECM) in a DMEM 31885029 cell culture medium (5.5 mM glucose) without serum but supplemented with 2% BSA, penicillin and streptomycin, 10 μM nicotinamide, 2.5 μg/ml transferrin, 6.7 ng/ml sodium selenite, and 50 μM of 2-mercaptoethanol as described earlier (8). For testing, cells were washed with PBS, followed by incubation with FFA (palmitate or oleate, Merck) at the concentration of 500 μM for 24 h in cell culture media with 1% fetal calf serum and FFA-free BSA as described earlier (8). The stock solutions of PA and OA (50 mM) were freshly prepared using 90% ethanol as a solvent at 62° (8). The ratio between FFA and added BSA was equivalent to a FFA/BSA molar ratio of 3.3 for INS-1E or 5 for EndoC-βH1 cells, representing the FFA/BSA ratios under pathological conditions (41). The calculated free, unbound FFA fraction represent approximately three- to five-times the concentration of unbound FFAs measured in the plasma of healthy lean individuals (42, 43). These calculated concentrations of free, unbound PA may correlate with the three-fold elevated concentrations of FFAs observed in individuals diagnosed with T2DM (44).

### Modification of the expression levels of SPL and SGPP1

**Fig. 1:**
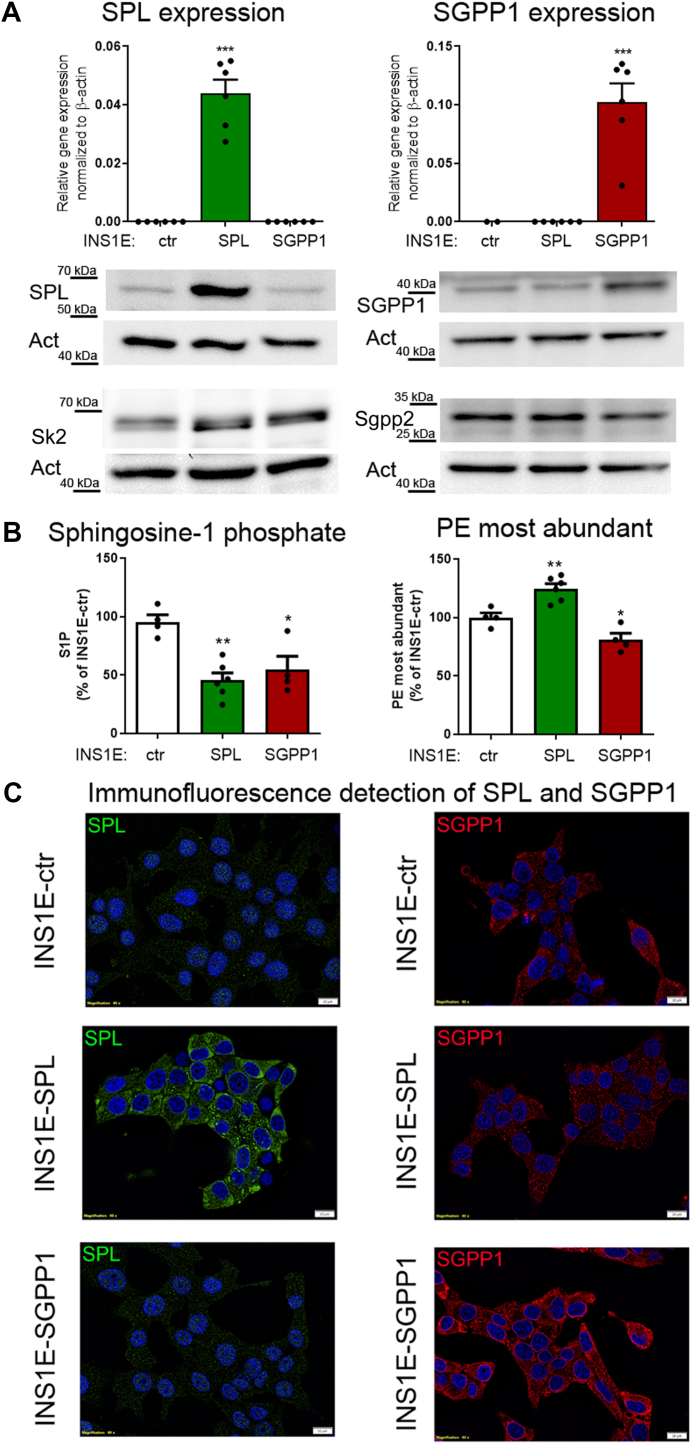
Overexpression of human SPL and human SGPP1 in insulin-secreting INS1E cells. Shown are (A) gene and protein expression of human SPL and human SGPP1 measured by qRT–PCR and Western blotting, (B) S1P concentration measured by ELISA and content of the most abundant PE (phosphatidylethanolamine, PE 34:1, 36:1, 36:2, and 38:4) measured by mass-spec; changes in S1P and PE content are shown as % of concentrations in INS1E-ctr cells, n = 4. (C) representative immunofluorescence pictures (from n = 2) after staining with antibodies for the detection of rat and human SPL and SGPP1 (*green*-SPL, *red*-SGPP1; note that since both primary antibodies were rabbit, a double immunostaining for parallel detection of SPL and SGPP1 in the same cells was not possible). Shown are Means ± SEM from four independent samples. ANOVA followed by Bonferroni, ∗*P* < 0.05, ∗∗*P* < 0.01, ∗∗∗*P* < 0.001 versus INS1E-control cells. The magnitude of SPL and SGPP1 overexpression was regularly checked during the entire time of cell culture.

The human SPL was stably overexpressed in insulin-secreting INS1E cells as described earlier (17). The human SGPP1 was overexpressed by a lentiviral transduction protocol using the pCLV-Ubic-MCS-IRES-SGPP1 (Sirion Biotech). Positive clones were selected using antibiotics (G418 for SPL, puromycin for SGPP1), and the gene and protein expression levels of SPL and SGPP1 were confirmed. Transfections with empty vectors did not affect INS1E cell viability or susceptibility to FFA (data not shown). For INS1E-ctr cells, we refer to the pooled data from untransfected (no selective antibiotic), pcDNA3-empty (mock, G418), and pCLV-Ubic-MCS-IRES-empty (mock, puromycin)-transfected INS1E cells. Our group recently published observations on the role of SPL in lipotoxicity in beta-cells (8). In the present study, we used for comparison the same cell clone as we described previously (8). However, these cells were generated using a SPL-GFP fusion construct, which results in a bright green fluorescence signal with SPL overexpression. Thus, for all experiments requiring fluorescence measurements of other parameters, we used cells transfected with an SPL construct lacking tagged GFP. Control experiments have shown that transfections with either the SPL-GFP or the SPL-without GFP constructs result in successful overexpression (C_T_ of 20–23 in real-time qRT-PCR) of the active enzyme at the similar level [(8) and Fig. 1]. EndoC-βH1-ctr and SGPP1-overexpressing human beta-cells were transiently transfected with validated Silencer®Select RNAi against human SPL (80 nM, assay S16965, Thermo Fisher Scientific) or a scramble siRNA (siQ, Ambion^TM^Silencer Negative Control, Thermo Fisher Scientific) using Lipofectamine RNAiMax in OptiMEM medium according to the protocol (8). Twenty–four hours after transfection cell culture medium was changed. Seventy-two hours post-transfection, FFA incubations were started as described above.

### Viability, caspase-3/7, and ATP assay

INS1E cells were seeded onto 96-well plates at a concentration of 30,000 cells/well and cultured for 48 h. Thereafter, cells were treated with FFAs for 24 h, and cell viability was determined by a microplate-based MTT assay [3-(4,5-dimethylthiazol-2-yl)-2,5-diphenyl tetrazolium bromide (Serva, Heidelberg, Germany)] as previously described (8, 17). The MTT assay is based on the conversion of MTT into formazan crystals by living cells and detects total mitochondrial activity, which correlates with the number of cells. In EndoC-βH1-ctr and SGPP1-overexpressing beta-cells, the sensitivity to FFA-induced apoptosis after knock-out of SPL was measured 24 h after FFAs incubation by a 96-well-based chemiluminescence Caspase-3/7-Glo assay (Promega) according to the manufacturer’s protocol. ATP content was measured after exposure to FFA by ATPLite assay, according to the manufacturer’s protocol (17).

### Oxidative stress assessment

To detect overall oxidative and nitrosative stress, INS1E cells were seeded onto 96-well black plates. Before addition of FFA, cells were pre-incubated with 10 μM dichlorodihydrofluorescein diacetate DCFDA-H_2_ (Thermo Fisher Scientific, Bremen, Germany) for 40 min at 37°C. Plates were analyzed at 480/520 nm excitation/emission using the fluorescence reader Synergy Mx multi-mode microplate reader (BioTek, Winooski, VT) as described earlier (8, 17). Each condition was analyzed at least in duplicate. Data were normalized to cell viability and expressed as a % of untreated cells. The subcellular hydrogen peroxide (H_2_O_2_) generation was estimated using fluorescence sensor HyPer proteins. Cells expressing HyPer proteins in cytoplasm (Hyper-Cyto), mitochondria (HyPer-Mito), or peroxisomes (HyPer-Peroxi) were seeded onto Mat-Tek glass bottom 35 mm dish (MatTek Corporation, Ashland). Cells were cultured for 48 h and afterwards exposed to FFAs for 24 h. Live cell imaging was performed using a CFP-YFP dual filter (excitation, 427 nm and 504 nm; emission, 520 nm) with a Olympus IX 81 inverted microscope system and the CellSens software (Olympus, Hamburg, Germany) for imaging and analysis. Shown are representative pictures from n = 3 individual experiments.

### RNA isolation, cDNA synthesis, and real-time RT-PCR

Total RNA from insulin secreting INS1E cell clones was obtained using the RNeasy kit (Qiagen, Hilden, Germany). The quality of the total RNA was verified by agarose gel electrophoresis. RNA was quantified spectrophotometrically at 260/280 nm. Thereafter, 2 μg of RNA were reverse transcribed into cDNA using a random hexamer primer (Life Technologies) and RevertAid H Minus M-MuLV reverse transcriptase (Thermo Fisher Scientific, Bremen, Germany). QuantiTect SYBR Green™ technology (Qiagen), which uses a fluorescent dye that binds only double-stranded DNA, was employed. The reactions were performed on a ViiA7 real-time PCR system (Life Technologies) with the following protocol: 50°C for 2 min, 95°C for 10 min, and 40 cycles comprising a melting step at 95°C for 15 s, an annealing step at 62°C for 60 s, and an extension step at 72°C for 30 s. The quality of reactions was controlled by the analysis of melting curves. Each sample was amplified as triplicate. Data normalization was performed against the geometric mean of the housekeeping gene β-actin. The primer sequences are given in supplemental Table S1.

### Western blot analyses

Cells were homogenized in ice-cold PBS containing protease inhibitors (Roche, Mannheim, Germany) using short bursts (Braun-Sonic 125 Homogenizer, Quigley-Rochester, Rochester, NY). Protein content was determined by the BCA assay (Pierce). For Western blotting, 20–40 μg of total protein was resolved by SDS-PAGE and then electroblotted onto membranes. Immunodetection was performed using specific primary antibodies (supplemental Table S2) as described (8). Pictures were captured by the INTAS chemiluminescence detection system (Intas Science Imaging Instruments, Göttingen, Germany). The intensity of bands was quantified through densitometry with Gel-Pro Analyzer version 4.0 software (Media Cybernetics, Silver Spring, MD).

### Immunofluorescence staining

For immunofluorescence staining, INS1E cells were seeded onto collagen-coated glass slides and incubated as described above. The immunofluorescence staining and detection was performed as described before (45). For SPL and SGPP1 detection, slides (n = 2) were incubated with primary antibodies (rabbit polyclonal anti-SPL (Santa Cruz) or rabbit polyclonal anti-SGPP1 (Thermo Fisher Scientific)), followed by secondary antibodies (anti-rabbit Alexa Fluor 488-conjugated IgG or anti-rabbit Alexa Fluor 647-conjugated IgG, both from Dianova, Hamburg). For a parallel detection of mitochondrial network changes and ceramide (n = 3), cells were incubated with the 250 nM Mitotracker-Red™ (Thermo Fisher Scientific, Bremen, Germany) in Krebs-Ringer buffer for 10 min at 37°C, following washing with PBS and an overnight fixation with 4% (w/v) paraformaldehyde in PBS (slides were kept in dark) (45). After fixation, cells were washed three times with PBS for 5 min. After a 20 min blocking in PBS with 0.1% Triton X-100 and 1% (v/v) BSA at room temperature (20°C), slides were washed again as above. The slides were incubated overnight at 4°C with mouse anti-Ceramide antibody (1:50 dilution from Biozol, Eching, Germany) and then washed three times with PBS. The cells were incubated with secondary antibodies for 1 h [Alexa Fluor 488-conjugated anti-mouse IgG, 1:200 dilution (Dianova, Hamburg, Germany)]. For nuclear counterstaining, 300 nM 4,6-diamidino-2-phenylindole (DAPI) was used for 5 min at room temperature. Slides were thereafter mounted with Mowiol (Merck, Darmstadt, Germany) plus 0.6% Dabco (Sigma Aldrich, Munich, Germany). Images were captured and analyzed using the CellSens software (Olympus, Hamburg, Germany) on the Olympus IX81 inverted microscope system.

### Analysis of LD content and size

Cells were seeded onto 6-well plates and incubated as described above. Cells were trypsinized and fixed in 1% paraformaldehyde for 15 min at room temperature. Thereafter, cells were stained with Oil Red O solution (Sigma-Aldrich, Munich, Germany) followed by DAPI staining and washed twice with PBS (8). LD formation was analyzed using the CellSens/Olympus IX81 inverted microscope system (40 × objective, Olympus, Hamburg, Germany) (n = 4–6 independent experiments). The area within the INS1E cell clones was quantified by the use of CellSens Software (Olympus, Hamburg, Germany) at 546 nm excitation and 580 nm emission. For each condition, five to seven randomly selected images (each containing 3 to 10 cells) were used to quantify the proportion of the LD area to the total cell area with the phase analysis module of the CellSens software. Additionally, the size of LDs was estimated by the measurements of cell perimeter and grouping cells into three categories (small, medium, and large). The mean number of small, medium, and large LDs per cell was calculated for each condition and presented as % of total number of LDs of all sizes.

### Visualization of neutral lipids and lipophagy

INS1E cells were seeded onto collagen-coated glass slides and incubated as described above. For a parallel detection of lysosomes and neutral lipids, cells were loaded with 250 nM LysoTracker™ Deep Red (Thermo Fisher Scientific, Waltham, Bremen, Germany) in Krebs-Ringer buffer for 10 min at 37°C, following washing with PBS and fixation with 4% (w/v) paraformaldehyde in PBS for 30 min at room temperature (slides were kept in dark). After fixation, cells were rinsed with PBS three times. After washing, cells were incubated with 1X HCS LipidTOX™ green neutral lipid stain (1:1,000 dilution, Thermo Fisher Scientific, Waltham, MA) at room temperature for 30 min. For nuclear counterstaining, 300 nM DAPI was used for 5 min at room temperature. Slides were thereafter mounted with Mowiol (Merck, Darmstadt, Germany) plus 0.6% Dabco (Sigma-Aldrich, Munich, Germany). Images were captured and analyzed using the CellSens software (Olympus, Hamburg, Germany) on the Olympus IX81 inverted microscope system. Shown are representative pictures of n = 3 individual experiments.

### Measurement of S1P

Samples were prepared using lysis buffer: 20 mM PIPES, 150 mm NaCl, 1 mM EGTA, 1% (v/v) Triton X-100, 1.5 mM MgCl2, 0.1% SDS, 1 mM sodium orthovanadate, 1× protease inhibitor mixture (without EDTA), pH 7. The S1P-ELISA (Echelon, ImTec Diagnostics, Antwerpen, Belgium) was performed according to the manufacturer’s instructions. The absorbance at 450 nm was measured, and the concentration of S1P in the samples was determined by comparison with the standard curve as described (17).

### Determination of cellular lipid peroxidation status

Lipid peroxidation was quantified by Bodipy 581/591 C11 (final concentration of 1 μM, 30 min, Thermo Fisher Scientific, Bremen, Germany) staining as described (46). The entire process was performed in the dark. Stained INS1E cells from the medium as well as from the dishes (after trypsinization) were collected by centrifugation at 700 × g for 3 min and resuspended in ice-cold PBS. After two washes, samples were measured using the CyFlow ML cytometer (Partec, Münster, Germany). For each sample, 20,000 events were acquired and analyzed by FlowJo software (Tree Star, Ashland, OR). Lipid peroxidation was defined as the ratio of the mean fluorescence in the FL-1 channel (488 nm/527 nm) and the FL-3 channel (488 nm/620 nm). Data are expressed as the fold change of lipid peroxidation of INS1E-ctr cells.

### Lipidomics

Lipids were extracted as previously described (47). Samples were mixed for 2 min after addition of methanol (190 μl) and of 10 μl internal standard mix (0.1 μM S1P; 0.3 μM Sph, Cer 15:0 LPC 17:0, PC 17:0/17:0, SM 17:0, 5 μM LPE 17:1, PE 17:0/17:0, (Avanti Polar Lipids Inc, Alabaster, AL), as well as 10 μM d31-palmitic acid, d9-oleic acid and d5-hexadecanal and 3 μM Ergosterol (Cayman, Ann Arbor, MI) in methanol). Then, samples were centrifuged (21,300 × g, 4°C, 5 min), and the supernatant was transferred into mass spectrometry sample vials and stored at −80°C until measurement.

Prior to measurement, 100 μl aliquots of the supernatant were mixed with methanol (70 μl), 30 μl dansyl hydrazine (75 mM in MeOH), 30 μl EDC∙HCl (525 mM in MeOH), and 30 μl pyridine (3 mM in MeOH) and vortexed for 2 h at room temperature. Following addition of formic acid (3 μl), samples were incubated for 30 min at room temperature and concentrated by vacuum centrifugation (1,450 rpm, 40°C, 1 h). Residues were dissolved in acetonitrile (100 μl), transferred into mass spectrometry vials, and stored at −80°C until measurement.

Chromatographic separation was performed on a LCMS-8050 triple quadrupole mass spectrometer (Shimadzu Duisburg, Germany) interfaced with a Dual Ion Source and a Nexera X3 Front-End-System (Shimadzu Duisburg, Germany). Chromatographic separation for S1P, sphingosine, and ceramides was performed with a 2 × 60 mm MultoHigh-C18 RP column with 3 μm particle size at 40°C. Mobile phases consisted of [A] methanol and [B] aq. Formic acid (1% v/v) and the following gradient settings were used: [A] increased from 10% to 100% over 3 min (B.curve = −2) and returned to 10% from 8.01 min to 10 min prior to next injection. MS settings for S1P and sphingosine were the following: Interface: ESI, nebulizing gas flow: 3 L/min, heating gas flow: 10 L/min, interface temperature: 300°C, desolvation temperature: 526°C, desolvation line temperature: 250°C, heat block temperature: 400°C, drying gas flow: 10 L/min. MS settings for ceramide and steroids were the following: Interface: APCI, nebulizing gas flow: 2.4 L/min, heating gas flow: 3 L/min, interface temperature: 300°C, desolvation temperature: 526°C, desolvation line temperature: 250°C, heat block temperature: 400°C, drying gas flow: 3 L/min. Flow rate was 0.4 ml/min. Data were collected using multiple reaction monitoring (MRM), and positive ionization was used for qualitative analysis and quantification. Standard curves were generated by measuring increased amounts of analytes (10 nM–5 μM S1P, Sph, Cer 14:0, 16:0, 18:0, 18:1, 20:0, 22:0, 24:0, 24:1) with internal standard (S1P-d7 = 0.1 μM; Sph-d7, Cer 15:0 = 0.3 μM). Injection volume of all samples was 10 μl. The following MRM fragment ions (positive mode) were used for quantification: m/z = 264 and 82 for S1P, m/z = 252 for sphingosine, and m/z = 264 for Cer.

Mobile phases for phosphoglyceride, sphingomyelin, and fatty acid/aldehyde measurement consisted of [A] 10 mM aq. ammonium formate + 0.01% (v/v) formic acid and [B] acetonitrile + 0.01% (v/v) formic acid/isopropanol (1:1). Phosphoglyceride and sphingomyelin profiling was performed with a 3 × 150 mm Accucore Polar Premium column with 2.6 μm particle size and the following gradient settings were used: 20% [B] from 0 min to 1 min, 20%–40% [B] from 1 min to 2 min, 92.5% [B] from 2 min to 25 min, 100% [B] from 26 min to 35 min, and return to 20% [B] from 35.1 min to 38 min prior next injection. Data were collected using MRM. Positive ionization was used for quantification and standard curves were generated by measuring increased amounts of analytes (0.1 μM–10 μM, LPC 14:0, 16:0, and 18:0; PC 32:0, 34:1, 34:2, 36:0, 36:4, 36:6, 38:4, and 40:6; LPE 16:0, 18:0; PE 16:0/18:1; SM 32:1, 34:1, 36:1, 42:1 and 42:2) with internal standards (3 pmol LPC 17:0, PC 17:0/17:0, SM 17:0, 30 pmol LPE 17:1, PE 17:0/17:0). Fatty acid/aldehyde profiling was performed with a 2 × 60 mm MultoHigh¬C8 RP column with 3 μm particle size at 40°C and the following gradient settings were used: 40%–5% [B] from 0 min to 9.5 min (B.Curve = −2), 5% [B] to 0% [B] from 9.5 min to 12.0 min, and equilibration from 12.01 min to 15 min prior next injection. Flow rate was 0.4 ml/min and injection volume was 3 μl. MRM fragment ion m/z = 171 in ESI(+) mode was used for quantification and standard curves were generated by measuring increased amounts of analytes (0.1 μM–30 μM 2-HDE, HDA; 1 μM–300 μM fatty acid 16:0, 18:1) Linearity of standard curves and correlation coefficients were obtained by linear regression analysis. All MS analyses were performed with software LabSolutions 5.114 and LabSolutions Insight (Shimadzu, Kyoto, Japan) and further processed in Microsoft Excel. Data are shown as Means ± SEM in pmoles/sample.

### Data analysis

All data are expressed as Means ± SEM. Statistical analyses were performed using the Prism analysis software (Graphpad, San Diego, CA) using *t* test or ANOVA followed by Bonferroni correction, with *P* < 0.05 considered statistically significant.

## Results

### Overexpression of S1P enzymes catalyzing S1P turnover in insulin-secreting INS1E cells

To further validate the role of intracellular S1P in lipotoxicity in pancreatic beta-cells, we used insulin-secreting INS1E cells. These cells abundantly express Sk2 and Sgpp2, while Sgpp1 and Spl are hardly detectable (8, 17, 24), making them a useful model for studying the consequences of an augmented SPL and SGPP1 expression. INS1E cells were genetically modified to overexpress human SPL (8, 17) or human SGPP1. The expression of SPL and of SGPP1 was significantly elevated as revealed by measurements at the level of mRNA (human SPL and SGPP1) and protein (Western blot and immunofluorescence) (Fig. 1A and C). The expression of Sk2 and Sgpp2 was differentially affected by SPL versus SGPP1 overexpression (Fig. 1A), suggesting that changes in intracellular S1P turnover capacity may affect the expression of sphingolipid pathway enzymes. Furthermore, we observed a strongly reduced S1P concentration in the cell lysates of INS1E-SPL cells (approx. 60% reduction) but somewhat smaller 40% decrease of S1P content in INS1E-SGPP1 cells than INS1E-ctr cells (Fig. 1B). Consistent with previous reports, our result indicates that both overexpressed enzymes were active with SPL being slightly more efficient in reducing the cellular S1P than SGPP1 (48), probably due to a stronger Sk2 and a weaker Sgpp2 expression in INS1E-SGPP1 cells enabling a partial regeneration of the cellular S1P pool. Since the concentration of hexadecenal was below the detection limit (see below), we estimated the capacity of SPL to generate the reaction products indirectly by the assessment of phosphatidylethanolamine (PE) levels. PE is generated by different pathways within cells. It has been shown, however, that the amount of PE is significantly affected by changes in the enzymatic activity of SPL (49) as only one enzymatic step is needed to generate PE from ethanolamine phosphate, the second reaction product of SPL. Indeed, PE levels were elevated in INS1E-SPL cells, whereas they were reduced in INS1E-SGPP1 cells as compared to INS1E-ctr cells (Fig. 1B) strongly indicating higher SPL and SGPP1 activities, respectively.

### Enhanced S1P turnover modulates the effects of FFA on sphingolipid metabolism in insulin-secreting INS1E cells

Because we observed compensatory changes in S1P metabolic enzyme expression in cells overexpressing SPL and SGPP1, we analyzed the impact of S1P turnover on sphingolipid pathway enzyme expression and sphingolipid metabolism under control conditions and in the presence of FFA. We exposed cells to the most physiologically relevant and abundant FFA, namely saturated PA (500 μM) or monounsaturated OA (500 μM) for 24 h. These concentrations of FFA are standard effective concentrations used in rodent beta-cells with well-described effects upon a 24 h exposure (8, 9, 39, 46).

**Fig. 2:**
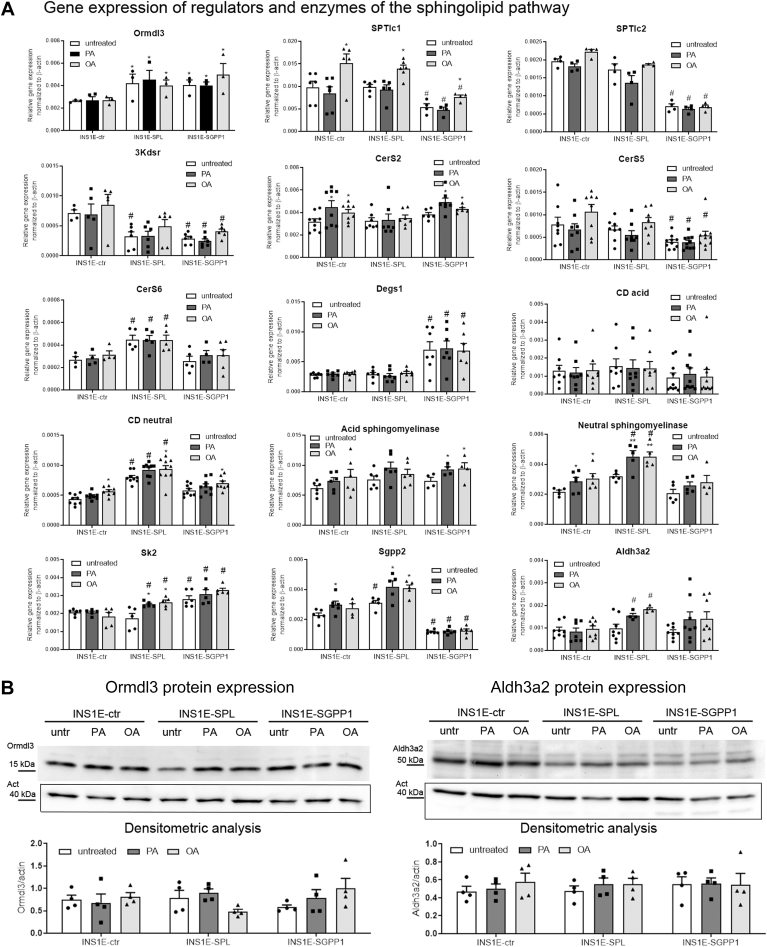
Effects of an increased S1P turnover on FFA-mediated changes of the expression of enzymes of the sphingolipid pathway in insulin-secreting INS1E cells. INS1E-ctr, -SPL and SGPP1 cells were incubated in the absence or presence of PA or OA (each of 500 μM) for 24 h. Thereafter (A) RNA was isolated, cDNA was synthesized and real-time PCR was performed. Shown are MEANS ± SEM from n = 4–8 independent experiments, each condition was measured in triplicates. (B) protein expression was analyzed by Western blotting, shown are representative blots and densitometry analysis of protein expression normalized to β-actin of n = 4 individual experiments. ANOVA followed by Bonferroni, ∗*P* < 0.05, ∗∗*P* < 0.01, ∗∗∗*P* < 0.001 versus untreated, #*P* < 0.05 versus INS1E-ctr cells treated in the same way.

The major regulation of sphingolipid de novo biosynthesis occurs post translationally via ORMDL proteins, of which ORMDL3 is abundantly expressed in beta-cells (Fig. 2). Overexpression of SPL or SGPP1 significantly increased the mRNA expression of Ormdl3, without any significant effect of FFA treatment (Fig. 2A). Former studies indicate that ORMDL3 gene and protein expression levels are not always concordant, which suggests that ORMDL3 might be differentially regulated on the transcriptional versus translational levels (50). Indeed, translation of Ormdl3 mRNA was not always congruent with the amount of transcript (Fig. 2A and B). As depicted in Figure 2B, Ormdl3 protein expression was somewhat lower in INS1E-SGPP1 than other untreated cells. FFA treatment did not significantly influence the translation of Ormdl3 transcript in any analyzed cell clones (Fig. 2B). This result prompted us to analyze possible adjustments of sphingolipid biosynthesis and degradation capacity that could contribute to FFA toxicity in these cells. We started with the serine palmitoyl transferase (SPT), the rate-limiting enzyme of de novo sphingolipid biosynthesis, which converts L-serine and palmitoyl-CoA to 3-oxosphinganine. We determined the mRNA level of both SPT long chain (SPTlc) subunits 1 and 2 required for enzymatic activity. The expression of SPTlc1 was not affected by PA but significantly upregulated by OA in all analyzed cell clones (Fig. 2). The expression of SPTlc1 was, however, significantly lower in INS1E-SGPP1 cells than in INS1E-ctr and INS1E-SPL cells (Fig. 2). SPTlc2 expression was not affected by FFA exposure but was strongly reduced by SGPP1 overexpression (Fig. 2). This result indicates a reduced rate of de novo sphingolipid biosynthesis in INS1E-SGPP1 cells. The mRNA expression of 3-ketodehydrosphingosine reductase catalyzing the reduction of 3-oxosphinganine to sphinganine was significantly lower in untreated INS1E-SPL and INS1E-SGPP1 cells than in control cells (Fig. 2), with a weak tendency of elevated expression following OA exposure (Fig. 2). N-acylation of sphinganine to dihydroceramide is catalyzed by three of the six known ceramide synthase isoforms, namely CerS2, CerS5, and CerS6, predominantly expressed in INS1E cells. The effects of SPL and SGPP1 overexpression as well as of FFA treatment on the mRNA expression of these enzymes are rather heterogenous. Thus, CerS2 expression was not affected neither by SPL nor by SGPP1 overexpression but was elevated by FFA treatment in INS1E-ctr and INS1E-SGPP1 cells. However, CerS5 expression was considerably reduced in INS1E-SGPP1 cells as compared to control and SPL overexpressing cell clones, while the expression of CerS6, which is responsible for ceramide formation in mitochondria, was significantly upregulated only in INS1E-SPL cells independently of FFA treatment (Fig. 2). On the other hand, the expression of sphingolipid-delta-4-desaturase (Degs1), catalyzing the formation of ceramide from dihydroceramide, was significantly higher in INS1E-SGPP1, but not affected by FFA (Fig. 2). Next, we analyzed the transcript amounts of two enzymes catalyzing ceramide hydrolysis in different cellular compartments at different pH optima. Acid ceramidase (CD), which is mainly localized to the lysosomes, was affected neither by overexpression of SPL and SGPP1 nor by FFAs treatment. Yet, the expression of neutral CD, which is active in various cellular membranes, was significantly increased by SPL overexpression as well as by OA treatment (Fig. 2). We then determined the amount of acid and neutral sphingomyelinase mRNA, as these two enzymes are known to generate ceramide from sphingomyelin, one of the most abundant sphingolipids in cellular plasma membranes (15). While expression levels of the lysosomal enzymes acid sphingomyelinase and acid CD were not affected by the different conditions, the expression of neutral sphingomyelinase was significantly increased by FFA treatment especially in SPL-overexpressing cells (Fig. 2). Similar to neutral CD, neutral sphingomyelinase was not affected in SGPP1-overexpressing cells independently of FFA treatment (Fig. 2). We then analyzed the expression of two enzymes directly involved in S1P metabolism. The expression of SK2, which phosphorylates sphingosine yielding S1P, was strongly elevated on the one hand by SGPP1 overexpression and on the other hand by PA and OA treatment but only of INS1E-SPL cells (Fig. 2). The expression of SGPP2, the predominant S1P phosphatase isoform in beta-cells, was significantly elevated in SPL overexpressing cells but considerably reduced in SGPP1 overexpressing cells (Fig. 2). PA treatment also increased SGPP2 expression in control and SPL overexpressing cells, whereas OA treatment had a similar increasing effect but only in INS1E-SPL cells (Fig. 2). Finally, we analyzed the expression of the aldehyde dehydrogenase 3 family member A2 (Aldh3a2), the enzyme that rapidly oxidizes hexadecenal, a rather toxic product of the SPL reaction, to form palmitoleic acid. Aldh3a2 was strongly expressed in all analyzed INS1E cell clones (Fig. 2A and B). Interestingly, FFA tended to elevate the Aldh3a2 protein expression in INS1E-SPL cells (Fig. 2B), indicating that under lipotoxic stress, INS1E-SPL cells might be equipped with a better hexadecenal detoxification capacity.

**Fig. 3:**
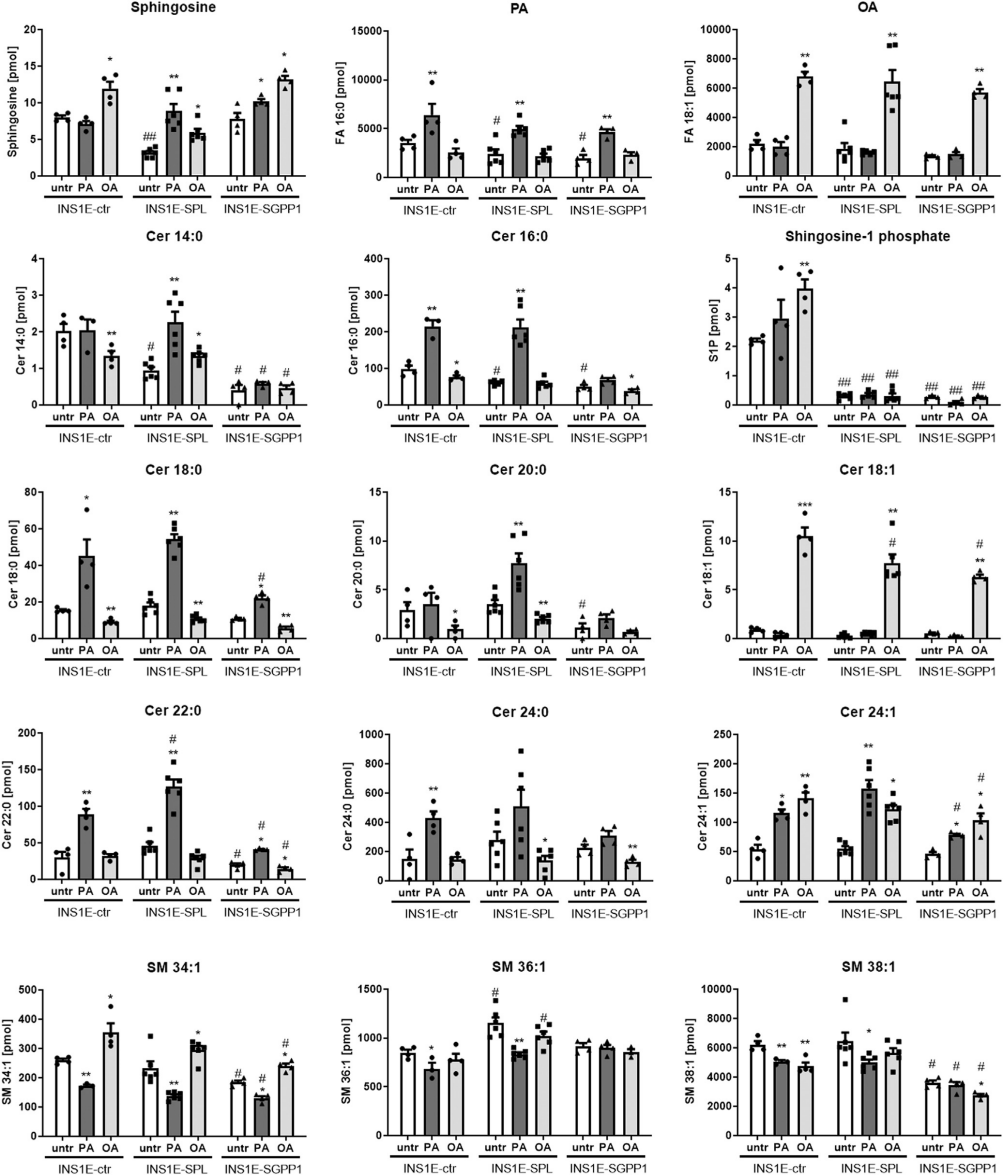
Effects of an increased S1P turnover in insulin-secreting INS1E cells on lipid species after exposure to FFA. INS1E cells were incubated in the absence or presence of PA or OA (500 μM of each) for 24 h. Thereafter, lipids (sphingosine, S1P, PA, OA, Cer = ceramide, SM = sphingomyelin) were extracted and analyzed by mass-spec as described in Methods. Shown are Means ± SEM from n = 3–6 independent experiments. ANOVA followed by Bonferroni, ∗*P* < 0.05, ∗∗*P* < 0.01, ∗∗∗*P* < 0.001 versus untreated, #*P* < 0.05 versus INS1E-ctr cells treated in the same way.

We then performed a lipidomic analysis to check whether and how the observed changes in the expression of sphingolipid pathway enzymes affected the amount of certain lipid species (Fig. 3). First, we measured PA and OA content and observed significantly lower levels in untreated INS1E-SPL and INS1E-SGPP1 as compared to control cells. As expected, incubation of cells with PA and OA, respectively, caused an increase of the respective FA (Fig. 3). The measurements of sphingosine revealed a significantly lower level in untreated INS1E-SPL cells as compared to control and SGPP1 cells (Fig. 3). While OA increased sphingosine content in all analyzed clones, PA affected sphingosine levels differentially. PA did not affect the sphingosine content in INS1E-ctr, while it tended to upregulate it in INS1E-SGPP1 cells (Fig. 3). Only in the INS1E-SPL cells, PA strongly enhanced sphingosine content (Fig. 3). S1P content was significantly increased by OA treatment in INS1E-ctr cells (Fig. 3). No significant effects of FFA incubations on S1P levels in INS1E-SPL or SGPP1 cells were detected (Fig. 3). Importantly, we were unable to detect hexadecenal in INS1E-ctr and SGPP1 cells, consistent with the low expression of SPL in these cells. Also, in untreated or FFA-treated INS1E-SPL cells, no hexadecenal could be detected, strongly indicating that the abundant expression of Aldh3a2 (Fig. 2) was able to efficiently remove the toxic aldehyde. Interestingly, we observed profound differences in the ceramide species profile in response to FFA between the clones examined. The content of various sphingomyelins was decreased in response to PA in INS1E-ctr and SPL cells and to a lesser extent in INS1E-SGPP1 cells (Fig. 3).

Together, our results show that both manipulated S1P metabolism and FFA treatment affect sphingolipid metabolism in an additive or independent manner. The observed changes suggest moreover, that the transcription of enzymes involved in sphingolipid metabolism is controlled by *i*) dietary FFA, *ii*) intracellular S1P levels, and/or *iii*) other bioactive sphingolipids closely associated with S1P and FFA metabolism.

### Upregulation of S1P recycling has opposite effects on beta-cell susceptibility to FFA to those caused by enhanced S1P degradation in insulin-secreting INS1E cells

**Fig. 4:**
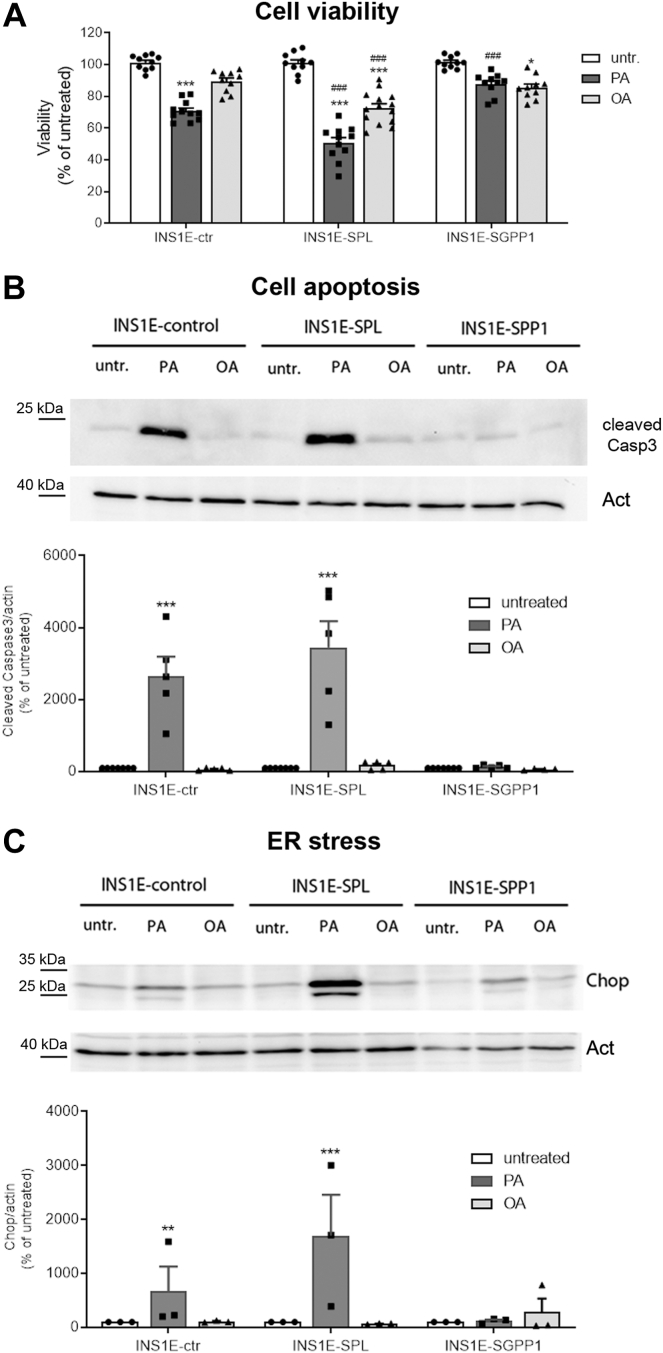
Effects of an increased S1P turnover in insulin-secreting INS1E cells on cell viability, apoptosis, and ER stress after exposure to FFA. INS1E cells were incubated in the absence or presence of PA or OA (500 μM of each) for 24 h. Thereafter, (A) cell viability was measured by MTT assay, (B) activation of caspase-3 was detected by Western blot analysis of cleaved caspase-3 protein expression, and (C) induction of Chop was detected by Western blot analysis. Shown are representative WBs and a densitometry analysis of protein expression detected in all experiments. Shown are Means ± SEM from n = 3–6 independent experiments, each condition was measured in triplicates. ANOVA followed by Bonferroni. ∗*P* < 0.05, ∗∗*P* > 0.01, ∗∗∗*P* < 0.001 versus untreated, ###*P* < 0.001 versus INS1E-ctr cells treated in the same way.

**supplemental Fig. S1:**
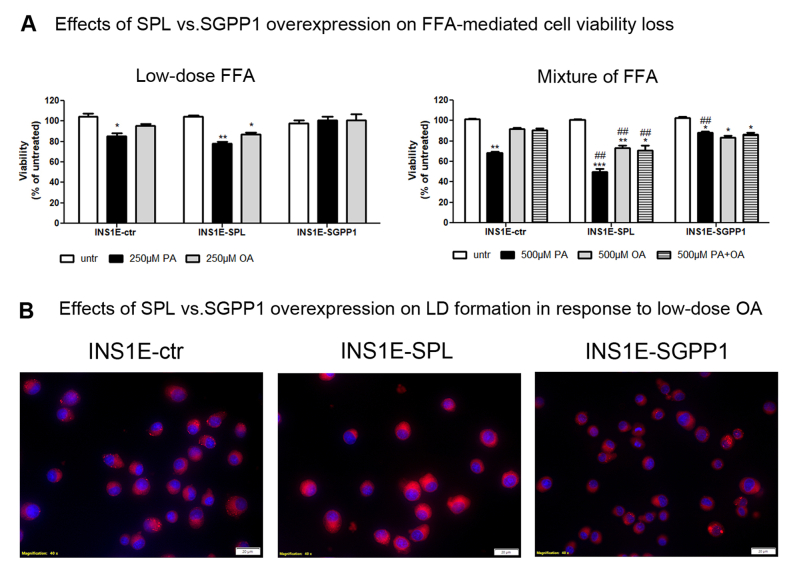
Effects of S1P turnover on cell viability and lipid droplet formation in INS1E beta-cells after exposure to low concentrations of FFA. INS1E cells were incubated in the absence or presence of PA and/or OA (low-dose: 250 μM of each, high dose and mixtures: 500 μM) for 24 h. Thereafter, (A) cell viability was measured by MTT assay, shown are Means ± SEM from n = 3–4 independent experiments, each conditions was measured in triplicates. ANOVA followed by Bonferroni, ∗*P* < 0.05 versus untreated. (B) lipid droplets (after exposure to 250 μM OA) were visualized by OilRed staining, shown are representative pictures of n = 2 individual experiments. Bars: 20 μm.

The analysis of cell viability confirmed a well-known toxic effect of PA (500 μM) and the lack of toxicity of OA (500 μM) in INS1E-ctr cells (Fig. 4A). In line with our earlier findings, SPL overexpression significantly potentiated PA-induced loss of cell viability. That was in contrast to the nearly complete lack of toxic effect of PA in INS1E-SGPP1 cells (Fig. 4A). Interestingly, the incubation with OA led to a significant decrease of cell viability both in INS1E-SPL and – to a lesser extent – in INS1E-SGPP1 cells (Fig. 4A). Similar, but slightly weaker effects of SPL versus SGPP1-overexpression were visible upon exposure to lower FFA concentrations (250 μM) and the mixture of PA+OA (500 μM) (supplemental Fig. S1). In the subsequent experiments, we decided to focus on the opposite effects of PA versus OA at the higher concentrations, the approach which allows a direct and clear comparison of a differential role of S1P turnover capacity on lipotoxicity in beta-cells indicative for effects related to diets enriched in saturated versus unsaturated FFA.

The MTT assay strongly depends on total mitochondrial activity, which mirrors the number of cells for most cell populations. To investigate whether the effects observed in the MTT assay correlated with induction of apoptosis, we employed caspase-3-cleaved assay. PA induced caspase-3 activation in INS1E-ctr and even more strongly in INS1E-SPL cells but failed to induce apoptosis in INS1E-SGPP1 cells (Fig. 4B). OA did not induce caspase-3 activation in INS1E-ctr and INS1E-SGPP1 cells but slightly increased caspase-3 cleavage in INS1E-SPL cells (Fig. 4B).

Finally, we studied the differential effects of SPL versus SGPP1 overexpression on the expression of Chop (C/EBP homologous protein), an ER stress marker that is induced by lipotoxicity in beta-cells and is believed to play an important role in beta-cell death (51). The fact that overexpression of SPL and SGPP1 did not increase Chop expression in untreated cells argues against their role as trigger of the ER stress response. Confirming earlier studies, we found that PA was a particularly strong inducer of Chop expression in control INS1E cells (Fig. 4C). The PA-mediated effects were potentiated by SPL overexpression in line with our earlier observations (8), but not by SGPP1 overexpression (Fig. 4C). The Chop expression was weakly induced by PA in INS1E-SGPP1 cells (Fig. 4C).

### Differential effects of enhanced S1P turnover on oxidative stress and identification of intracellular sources of ROS in insulin-secreting INS1E cells

**Fig. 5:**
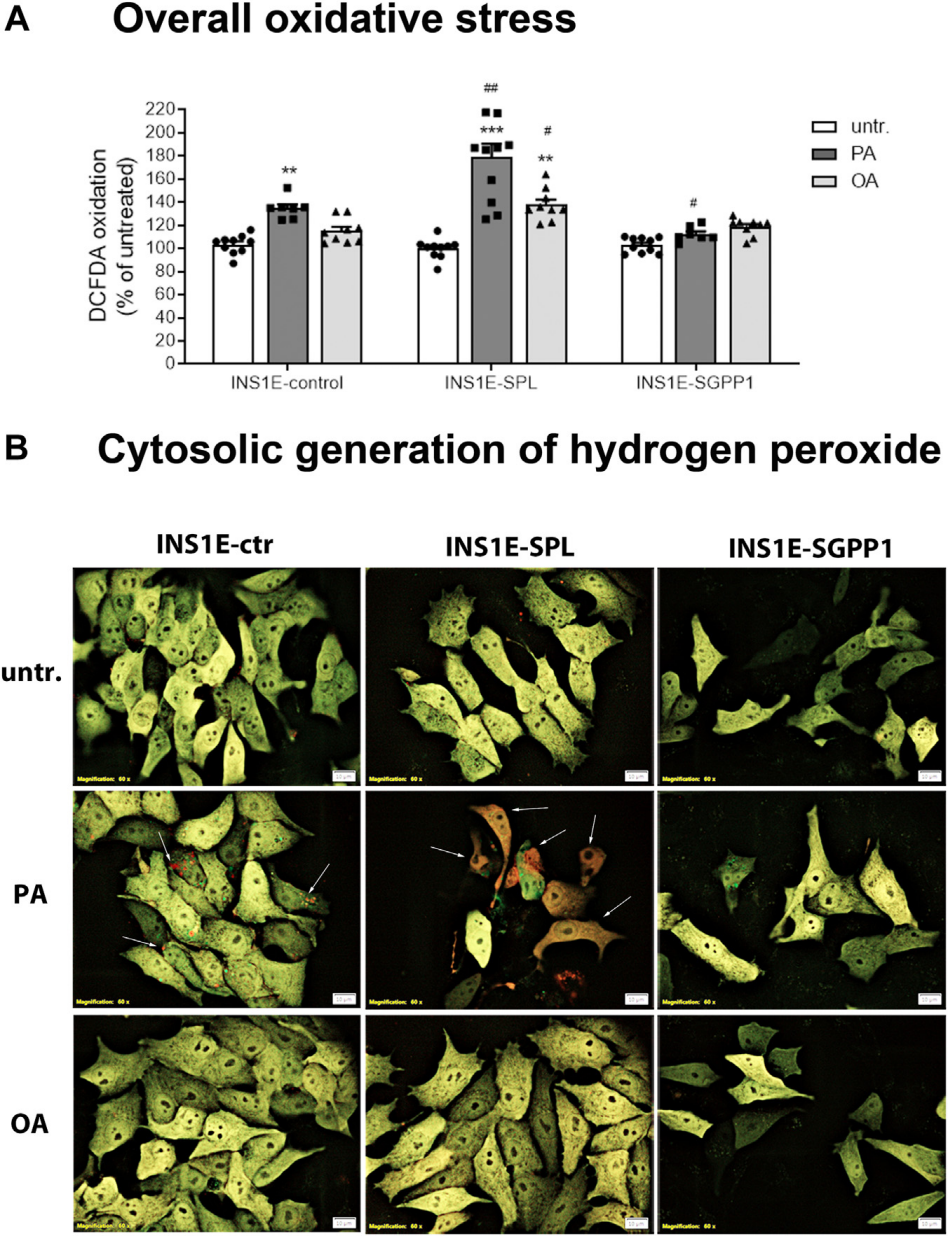
Effects of enhanced S1P turnover and FFA on overall oxidative stress and cytosolic hydrogen peroxide production in insulin-secreting INS1E cells. INS1E cells were incubated in the absence or presence of PA or OA (500 μM of each) for 24 h. (A) overall oxidative stress was measured by DCFDA method. (B) cytosolic hydrogen peroxide generation was estimated by expressing the hydrogen peroxide-sensitive fluorescence sensor HyPer-Cyto protein in the cytosolic compartment of cells and evaluation of fluorescence shift by the CellSens software at the Olympus fluorescence microscope (representative pictures from n = 3 individual experiments), Bars: 10 μm. Shown (A) are Means ± SEM from n = 3–6 independent experiments, each condition was measured in triplicates ANOVA followed by Bonferroni, ∗∗*P* < 0.01, ∗∗∗*P* < 0.001 versus untreated, #*P* < 0.05, ##*P* < 0.01, versus INS1E-ctr cells treated in the same way.

Next, we investigated the oxidative stress response, which is believed to play a crucial role in the toxicity of lipids in beta-cells (2, 5, 9, 10, 46, 52, 53, 54). We analyzed the overall oxidative stress using a robust DCFDA-based assay, which enables the detection of increased generation of a variety of reactive oxygen species (ROS). A significant induction of oxidative stress in INS1E-ctr cells exposed to PA, but not to OA, was detected (Fig. 5A). Interestingly, SPL overexpression was associated with a significantly stronger induction of ROS generation in both PA-and OA-treated cells, as compared to INS1E-ctr cells (Fig. 5A). Neither PA nor OA induced oxidative stress in INS1E-SGPP1 cells (Fig. 5A). In line with these observations, we observed an induction of H_2_O_2_ generation in response to PA in all analyzed cell clones using the hydrogen peroxide-specific fluorescence sensor protein HyPer expressed in the cytosolic cell compartment, which was, however, hardly detectable in INS1E-SGPP1 cells. (Fig. 5B). The fluorescence emitted from HyPer sensor protein changes from green (low H_2_O_2_) to red (high H_2_O_2_ concentration) upon generation of H_2_O_2_. In INS1E-ctr cells, we observed some areas of yellow/orange fluorescence in cytoplasm with a dot-like pattern of red fluorescence upon exposure to PA (Fig. 5B). In INS1E-SPL cells treated with PA, big areas of cytoplasm were red indicating that H_2_O_2_ concentration increase was higher than in INS1E-ctr cells and that its distribution was much more widespread throughout the entire cells (Fig. 5B). In response to OA, a minor increase in H_2_O_2_ formation was observed only in INS1E-SPL cells expressing the HyPer-Cyto protein (Fig. 5B).

**Fig. 6:**
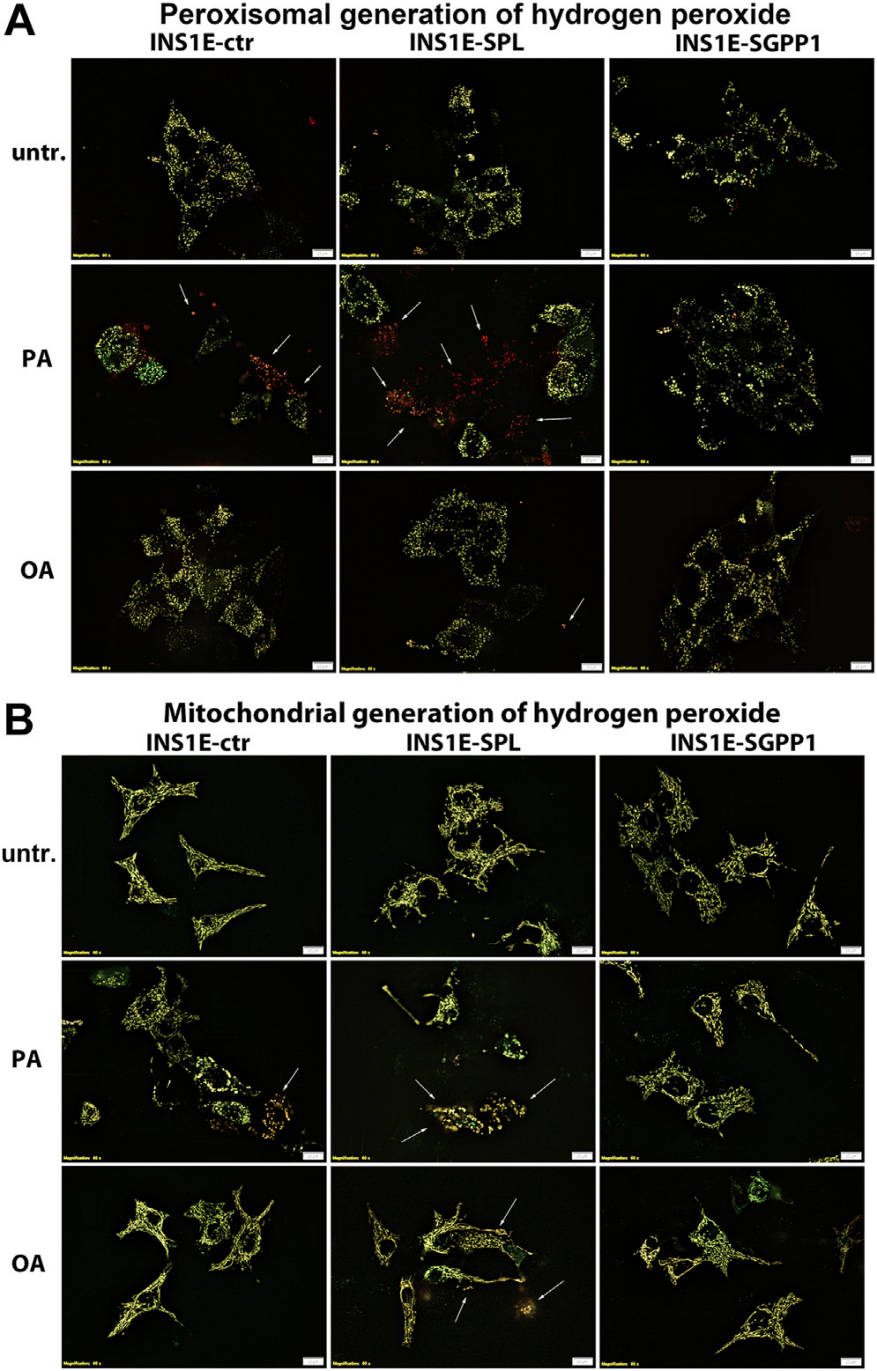
Effects of enhanced S1P turnover and FFA on peroxisomal and mitochondrial hydrogen peroxide production in insulin-secreting INS1E cells. INS1E cells were incubated in the absence or presence of PA or OA (500 μM of each) for 24 h. Peroxisomal hydrogen peroxide generation was estimated by expressing the fluorescence sensor HyPer-Peroxi (A) and mitochondrial by expressing HyPer-Mito (B) sensor proteins and evaluation of fluorescence shift by the CellSens software at the Olympus fluorescence microscope (representative pictures from n = 3 individual experiments). Arrows depict representative cells with higher red HyPer protein fluorescence. Bars: 10 μm.

To identify the subcellular sources of H_2_O_2_ generation, we expressed the HyPer sensors in peroxisomes and in mitochondria. Peroxisomes are considered the main source of H_2_O_2_ generation upon PA exposure in beta-cells. As anticipated, a strong induction of peroxisomal H_2_O_2_ generation was detected in response to PA in INS1E-ctr cells, which was further potentiated by SPL overexpression (Fig. 6A). In INS1E-SPL cells incubated with OA, a slightly increased red fluorescence of the HyPer-Peroxi protein was visible (Fig. 6A). In INS1E-SGPP1 cells, no induction of peroxisomal H_2_O_2_ generation was observed (Fig. 6A).

FFA are believed to mildly elevate mitochondrial H_2_O_2_ formation as a result of their mitochondrial metabolism, but not related to any toxic effect, with the exception of very long chain FFAs (5). This observation was confirmed in our study in INS1E-ctr cells (Fig. 6B). Interestingly, we observed increased red fluorescence in the HyPer-Mito protein expressed in INS1E-SPL cells exposed to PA and to a lesser extent also in INS1E-SPL cells incubated with OA (Fig. 6B), indicating high concentrations of H_2_O_2_ under these conditions. Moreover, the pattern of the HyPer-Mito staining was suggestive of mitochondrial damage in FFA-treated INS1E-SPL cells. Again, no increase in mitochondrial H_2_O_2_ formation was detected in INS1E-SGPP1 cells (Fig. 6B).

### Turnover of intracellular S1P affects mitochondrial stress and ceramide generation in response to FFA in insulin-secreting INS1E cells

**Fig. 7:**
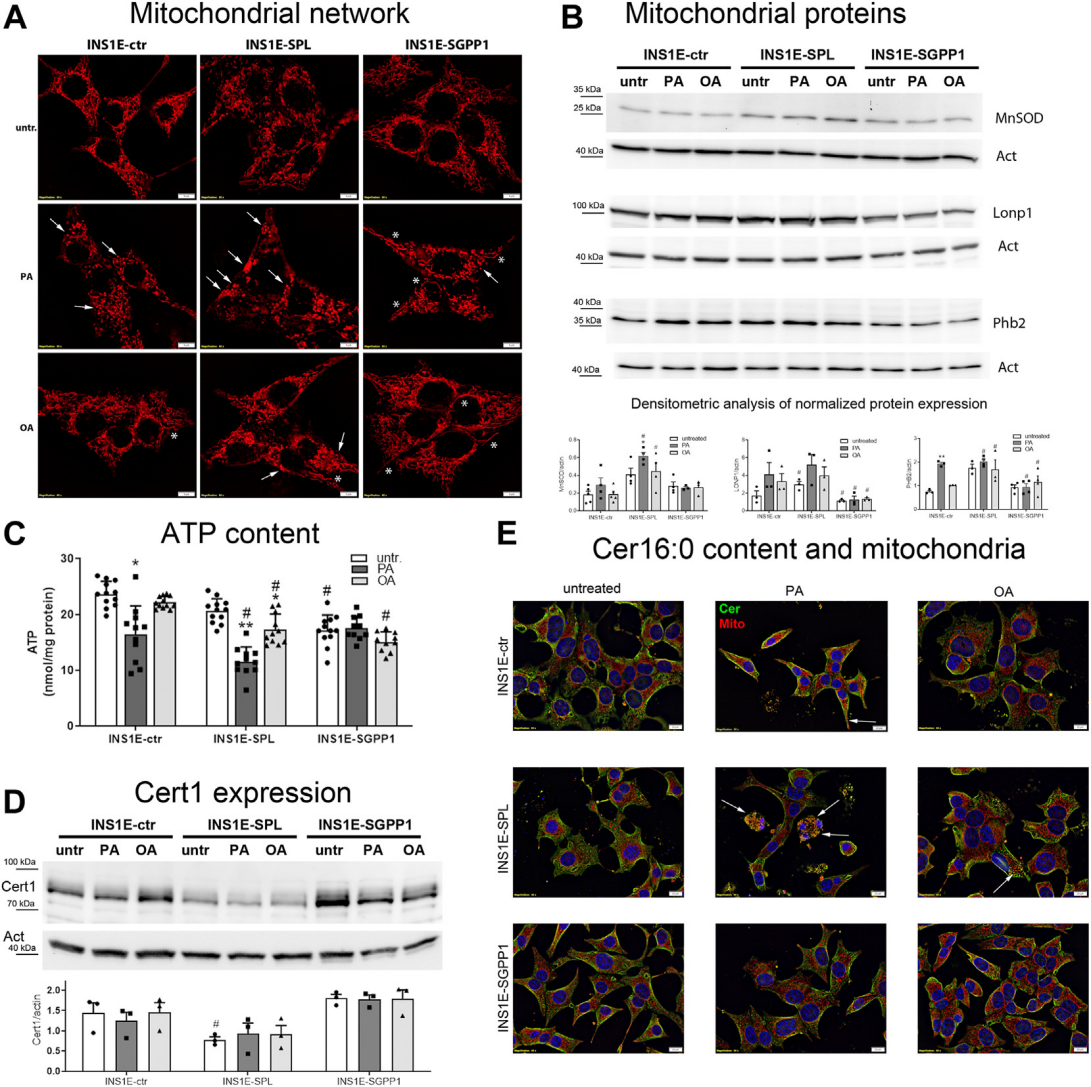
Effects of S1P turnover and free fatty acids on mitochondrial network, stress, and metabolic activity in insulin-secreting INS1E cells. INS1E cells were incubated in the absence or presence of PA or OA (500 μM of each) for 24 h. Thereafter, (A) mitochondrial network was analyzed after incubation with Mitotracker Deep Red™, Bars: 5 μm, (B) the expression of proteins important for mitochondrial stress was measured by Western blot, (C) ATP content was measured by ATPlite assay, shown are Means ± SEM from n = 4 independent experiments, each conditions was measured in triplicates. (D) Cert1 protein expression was analyzed by Western blot and (E) cellular distribution and content of ceramide (green) was performed by immunofluorescence in parallel with the MitoTracker Deep Red™ staining (red), Bars: 10 μm. Arrows depict the areas of ceramide and mitochondria colocalization (yellow). Shown are representative pictures of n = 3–5 independent experiments. Densitometry analyses of protein expression normalized to β-actin are shown as Means ± SEM from n = 3–5 independent experiments. ANOVA followed by Bonferroni, ∗*P* < 0.05, ∗∗*P* < 0.01, ∗∗∗*P* < 0.001 versus untreated, #*P* < 0.05, versus INS1E-ctr cells treated in the same way.

Since we observed signs of mitochondrial network fragmentation in FFA-exposed INS1E-SPL cells and an elevated H_2_O_2_ formation was detected, we further explored the mitochondrial network and mitochondrial stress markers. Using the MitoTracker Deep Red™ staining technique, we analyzed changes in the mitochondria network structure in response to FFA (Fig. 7A). PA induced the formation of round-shaped mitochondria in INS1E-ctr cells, however in a much stronger manner in INS1E-SPL cells, in which large aggregates of mitochondria could additionally be detected (Fig. 7A). Exposure to OA of INS1E-SPL cells caused milder effects but also resulted in the defective mitochondrial network appearance (Fig. 7A). In contrast, the mitochondrial network was enlarged and elongated in untreated and FFA-treated INS1E-SGPP1 cells.

Next, we analyzed the expression of mitochondrial proteins that could be involved in the regulation of oxidative stress, mitochondrial function, as well as in mitochondrial unfolded protein response (UPR^mt^). Interestingly, the expression of MnSOD, the mitochondrial isoform of superoxide dismutase, was significantly higher in INS1E-SPL cells than in other analyzed cell clones, and additionally enhanced by PA (Fig. 7B). The expression of Lonp1, a mitochondrial ATP-dependent serine protease that mediates the selective degradation of misfolded, unassembled, or oxidatively damaged polypeptides, and which is induced by oxidative stress, was increased in INS1E-ctr cells by FFA treatment (Fig. 7B). INS1E-SPL cells were characterized by an abundant basal expression of Lonp1, which was upregulated by FFA (Fig. 7B). A significantly lower expression of Lonp1 was detected in INS1E-SGPP1 cells. Prohibitin 2 (Phb2), an inner mitochondrial membrane protein that is involved in protection against oxidative damage, regulation of mitophagy, and ATP biosynthesis, was increased by PA and to a lesser extent by OA in INS1E-ctr cells (Fig. 7B). Confirming our earlier observations (17), we detected a higher basal expression of Phb2 in INS1E-SPL cells as compared to INS1E-ctr cells with no additional effect upon FFA treatment (Fig. 7B). In INS1E-SGPP1 cells, the expression of Phb2 protein was significantly lower than in INS1E-SPL cells, and it was not influenced by exposure to FFA (Fig. 7B).

The observed changes in mitochondrial network formation and mitochondrial proteins indicate an activation of mitochondrial stress response after treatment with FFA particularly in INS1E-SPL cells, whereas overexpression of SGPP1 obviously protected the cells against these deleterious effects. Mitochondrial stress results in a loss of adequate ATP generation. Indeed, in cells exposed to FFA for 24 h, PA decreased ATP content (by around 25%) in INS1E-ctr cells and even more in INS1E-SPL cells (by around 40%) (Fig. 7C). While OA did not affect ATP content significantly in INS1E-ctr cells, it led to a significant drop in ATP content in INS1E-SPL cells by about 15% (Fig. 7C). INS1E-SGPP1 cells were characterized by a lower basal ATP content than INS1E-ctr or INS1E-SPL cells, which was, however, not influenced by FFA (Fig. 7C).

Mitochondrial function and survival have been shown to be regulated by formation and accumulation of proapoptotic ceramide species (55). In INS1E-SPL cells treated with FFA, we observed higher neutral sphingomyelinase and CerS6 expression levels that could favor the observed ceramide increase, particularly in the mitochondria. Ceramide is synthesized at the ER and translocated by CERT1 to the Golgi compartment for conversion to sphingomyelin (56, 57). A disturbed or lower capacity of ceramide transport to the Golgi could favor ceramide transport to mitochondria and its intramitochondrial accumulation. Yet, the expression of Cert1 in INS1E-ctr cells was not affected by FFA (Fig. 7D). Interestingly, in INS1E-SPL cells, a significantly lower expression of Cert1 was observed, which was not further influenced by FFA (Fig. 7D). The expression of Cert1 in INS1E-SGPP1 cells was high and, similarly to other cell clones, not affected by FFA (Fig. 7D). Finally, using a well-established immunofluorescence ceramide staining (green) together with the MitoTracker staining (red), we visualized ceramide content of cells exposed to FFA with a particular focus on its mitochondrial localization (Fig. 7E). Ceramide content was increased in response to PA in INS1E-ctr and INS1E-SPL cells, but not in INS1E-SGPP1 cells (Fig. 7E). Interestingly, in INS1E-SPL cells, we observed that ceramide tended to accumulate in the vicinity of mitochondria, while in INS1E-ctr cells, such a colocalization was rather rarely observed (Fig. 7E). A colocalization of ceramide and mitochondria was observed in OA-treated INS1E-SPL cells, however to a lower extent than in PA-treated cells (Fig. 7E).

Together, these results indicate that lipid distribution and use in beta-cells exhibiting an increased irreversible S1P degradation capacity is remarkably distinct from those with enhanced S1P recycling, significantly impacting mitochondrial dysfunction in response to FFA.

### Distinct effects of S1P recycling versus degradation on LDs formation, but similar on lipid peroxidation in insulin-secreting INS1E cells

Since we observed a distinct lipid distribution pattern in cells overexpressing SPL versus SGPP1, in the next step, we analyzed the lipid storage capacity of cells treated with FFA. During LD biogenesis, neutral lipids (e.g., triglycerides) accumulate within the ER bilayer, with rising concentrations leading to the formation of LD. An insufficient capacity of LD formation may lead to lipid overload of the ER, a process that can contribute to ER stress induction. The LD biogenesis is a complex process, for which the expression of several specific proteins, regulating bud formation, and lipid transfer, is crucial (58, 59). First, we assessed the expression of seipin, Dgat2, and Plin proteins that are essential for LD formation and function (58, 59, 60).

**Fig. 8:**
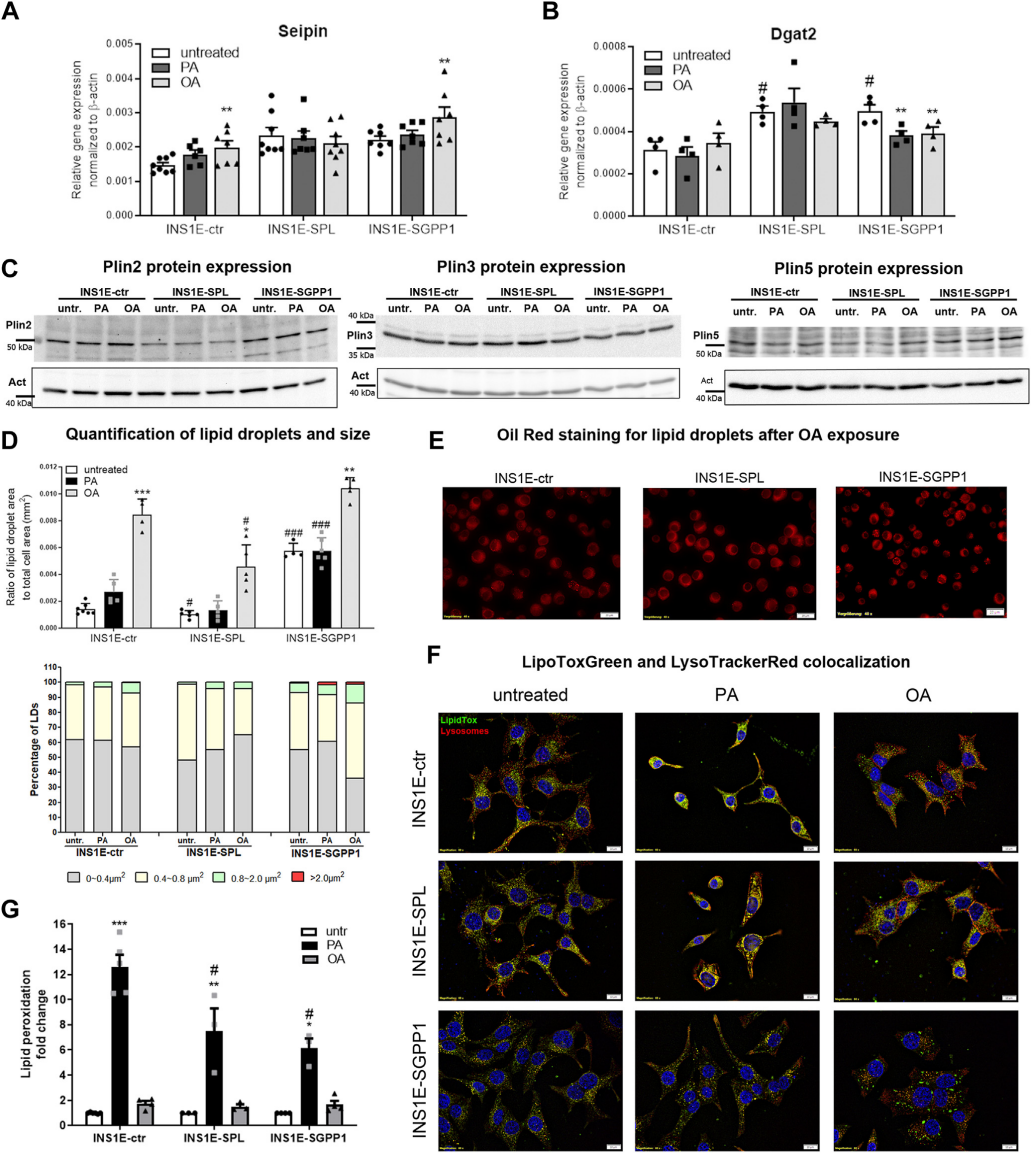
Effects of S1P turnover and free fatty acids on lipid droplet formation, content, size, and autophagy as well as on lipid peroxidation in insulin-secreting INS1E cells. INS1E cells were incubated in the absence or presence of PA or OA (500 μM of each) for 24 h. Shown are (A) the gene expression of seipin by qRT-PCR, (B) gene expression of Dgat2 by qRT-PCR (Means ± SEM from n = 4–8 independent experiments), (C) representative Plin2, Plin3, and Plin5 WBs from four independent experiments, (D) quantification of the number and size of lipid droplets after exposure FFA from n = 4–7 independent experiments, (E) lipid droplets detection by OilRed staining, a representative picture from n = 4–7 independent experiments, Bars: 20 μm, (F) colocalization of lipids (LipidTox Green) with lysosomes (LysoTracker Red), Bars: 10 μm, (G) lipid peroxidation, Means ± SEM from four independent experiments. ANOVA followed by Bonferroni, ∗*P* < 0.05, ∗∗*P* < 0.01, ∗∗∗*P* < 0.001 versus untreated, #*P* < 0.05, ###*P* < 0.001 versus INS1E-ctr cells treated in the same way.

The expression of seipin was unaffected in INS1E-SPL cells while significantly elevated only in OA-treated INS1E-ctr and SGPP1 overexpressing cells (Fig. 8A). The basal expression of Dgat2, which catalyzes the synthesis of triacylglycerol (the main cellular neutral lipid) and interacts with LD at the ER side, was significantly higher in both SPL and SGPP1 overexpressing cells (Fig. 8B). However, treatment with FFA significantly reduced its expression in INSIE-SGPP1 cells but had no additional effect in INSIE-SPL cells. (Fig. 8B).

Apart of Plin2 and Plin3, known to be abundantly expressed in INS1E cells, we also detected Plin5 (Fig. 8C), in line with earlier reports (61). Consistent with our previous data, we found a significantly lower expression of Plin2 in INS1E-SPL cells (Fig. 8C). In contrast, INS1E-SGPP1 cells were characterized by an abundant Plin2 expression (Fig. 8C). As shown in Figure 8C, no striking modifications of Plin3 expression were detectable (Fig. 8C). Plin5 expression, on the other hand, was downregulated in SPL overexpressing cells and also in control cells treated with PA (Fig. 8C).

Next, we analyzed LD generation by Oil Red O staining and observed a strong induction of LD formation in response to OA in INS1E-ctr cells, as shown by an increased ratio of LD area/total cell area and by the presence of multiple LD puncta in cells (Fig. 8D and E). A significantly lower number of LDs in INS1E-SPL cells, but a significantly higher number of LDs in INS1E-SGPP1 cells, was detected after exposure to OA (Fig. 8D). These effects were dose-dependent (supplemental Fig. S1). Interestingly, in untreated and PA-treated INS1E-SGPP1 cells, we observed the presence of a high number of LDs (Fig. 8D). We then analyzed the ratio of very small, small, medium, and large LDs in cell clones exposed to FFA (Fig. 8D and E). Former studies proposed that the expression pattern of specific Plin proteins like lipid composition might be involved in the regulation of LD size (42). The presence of small LDs has been shown to correlate with increased lipotoxicity in various cell types, while large LDs enable an efficient storage of cellular lipids (40, 61, 62). In line with the expression profile of LD proteins, untreated INS1E-ctr cells displayed around 60% LDs of very small/small size and 40% of medium/large size (Fig. 8D). While this proportion was not significantly changed upon exposure to PA, OA exposure resulted in accelerated formation of large LDs (Fig. 8D and E). In contrast, INS1E-SPL cells exhibited an increase of very small LDs upon exposure to PA and also to OA (Fig. 8D and E). Interestingly, INS1E-SGPP1 cells were rich in medium size LDs under basal conditions, and the number of very small LDs was significantly decreased in OA-treated cells (Fig. 8D). Additionally, we observed the presence of large LDs in PA and even more in OA-treated INS1E-SGPP1 cells (Fig. 8D and E).

We extended these observations by employing the LipidTox combined with the LysoTracker staining, to establish whether LD removal by lysosomes could also be causative for the lower LD number observed in INS1E-SPL cells. The LipidTox staining allows the detection of neutral lipid accumulation in cells and has an extremely high affinity for neutral lipid droplets. Using this method, we observed a diffused neutral lipid staining, with a mild colocalization with lysosomes, in untreated INS1E-ctr and INS1-SPL cells (Fig. 8F). In untreated INS1E-SGPP1 cells, the lipid staining was less diffuse, and well-defined LDs were detected (Fig. 8F). Following PA incubation, we noticed a more intense staining of lipids with a minimal LD presence and spatial distribution of lysosomes in INS1E-ctr cells. In contrast, a strong correlation of lipid and lysosome staining was visible in PA-treated INS1E-SPL cells, however, without LD-like structures and a high condensation of lysosomal network, suggesting activation of the lysosomal stress response (Fig. 8F). The incubation with PA did not increase the cytosolic lipid staining, but instead led to LD formation in INS1E-SGPP1 cells (Fig. 8F). In OA-treated INS1E-ctr cells, we observed a predominant LD presence and a weak overall and diffused lipid staining. Around 50% of LDs colocalized with lysosomes, indicating their functional turnover and an intact lysosomal network. The distribution of LipidTox staining in OA-treated INS1E-SPL cells was different; only a minor number of LDs, mainly of a smaller size, together with a diffused staining of the cytoplasmic compartment were evident (Fig. 8F). These small LDs colocalized with the lysosomal marker, indicating a high activity of lipophagy (Fig. 8F). In INS1E-SGPP1 cells, OA treatment increased the number of LDs, and many large LDs were distinguished (Fig. 8F). In contrast to INS1E-ctr and INS1E-SPL cells, the colocalization with the lysosomal tracker was rather scarce, indicating a lower rate of lipophagy (Fig. 8F). Apparently, SGPP1 overexpression improves lipid storage capacity in INS1E cells, whereas the opposite is true for SPL overexpression.

**supplemental Fig. S2:**
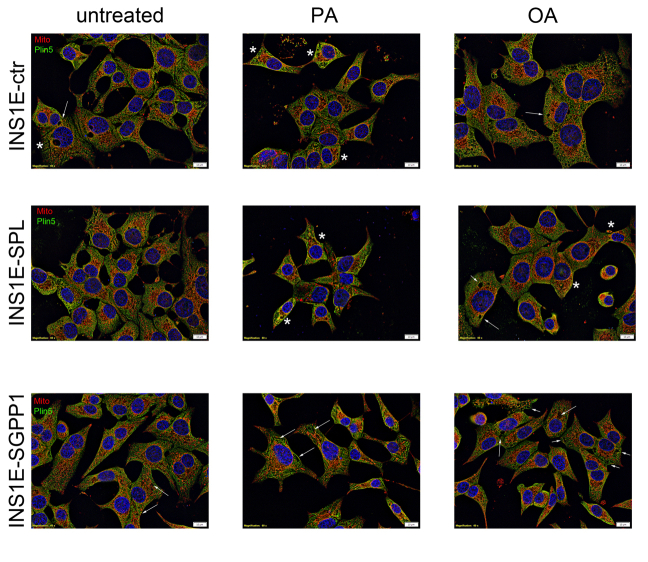
Effects of S1P turnover on the colocalization of Plin5 with mitochondria in INS1E beta-cells. INS1E cells were incubated in the absence or presence of PA or OA (500 μM of each) for 24 h. Thereafter, mitochondrial network was analyzed after incubation with Mitotracker Deep Red^TM^ (red) and cellular distribution Plin5 (green) was analyzed. Shown are representative pictures of n = 3 independent experiments. Asterixes: colocalization of Plin5 with mitochondria, arrows: Plin5-coated spherical structures not colocalized with mitochondria. Bars: 10 μm.

Since we detected ceramide accumulation in mitochondria along with impaired mitochondrial functions in FFA-treated INS1E-SPL cells, we performed a double-immunostaining of mitochondria with Plin5, an LD protein that has been shown to be involved in LD–mitochondria interaction and lipid transfer into mitochondria (supplemental Fig. S2). We observed Plin5 staining with spherical structures of various sizes that sometimes colocalized with mitochondria (supplemental Fig. S2). In PA-treated INS1E-ctr cells, the presence of Plin5-mitochondria costaining was detected (supplemental Fig. S2). In OA-treated INS1E-ctr cells, a large number of Plin5-LD structures was visible, but only a minority of small size LD-Plin5 structures colocalized with mitochondria (supplementary Fig. S2). In INS1E-SPL cells, a lower number of Plin5-LD structures was detected, in line with a lower Plin5 expression, but an increased co-staining of Plin5 and mitochondria was observed after exposure to PA as well as to OA (supplemental Fig. S2). In INS1E-SGPP1 cells, most of the Plin5-LDs did not colocalize with mitochondria (supplemental Fig.S2). Note that colocalization of Plin5 and mitochondria was specific, while the double immunofluorescence staining of Plin3 with mitochondria did not result in any colocalization (data not shown). These data are indicative of an increased lipid transfer from LDs to mitochondria in SPL overexpressing beta-cells that could cause an increased mitochondrial metabolism contributing to ROS formation. On the other hand, a decreased Plin5/mitochondria colocalization observed in INS1E-SGPP1 cells could be partially responsible for a lower ATP content, but a lack of oxidative stress in mitochondria in these cells.

To assess an impact of the observed lower capacity of lipid storage on the rate of lipid peroxidation, which is thought to correlate with PA toxicity in beta-cells (46), we performed a Bodipy 581/591 C11 staining (Fig. 8G). As expected, we observed a strong induction of lipid peroxidation in INS1E-ctr cells treated with PA, and no significant induction after exposure to OA (Fig. 8G). Surprisingly, both INS1E-SPL and INS1E-SGPP1 cells were protected against lipid peroxidation in response to FFA, with a slightly more efficient effect in SGPP1 overexpressing cells (Fig. 8G). Thus, an increased toxicity of FFA observed in INS1E-SPL cells did not depend on lipid peroxidation.

### S1P turnover influences the sensitivity to FFA and lipid droplets formation in human EndoC-βH1 beta-cells

**Fig. 9:**
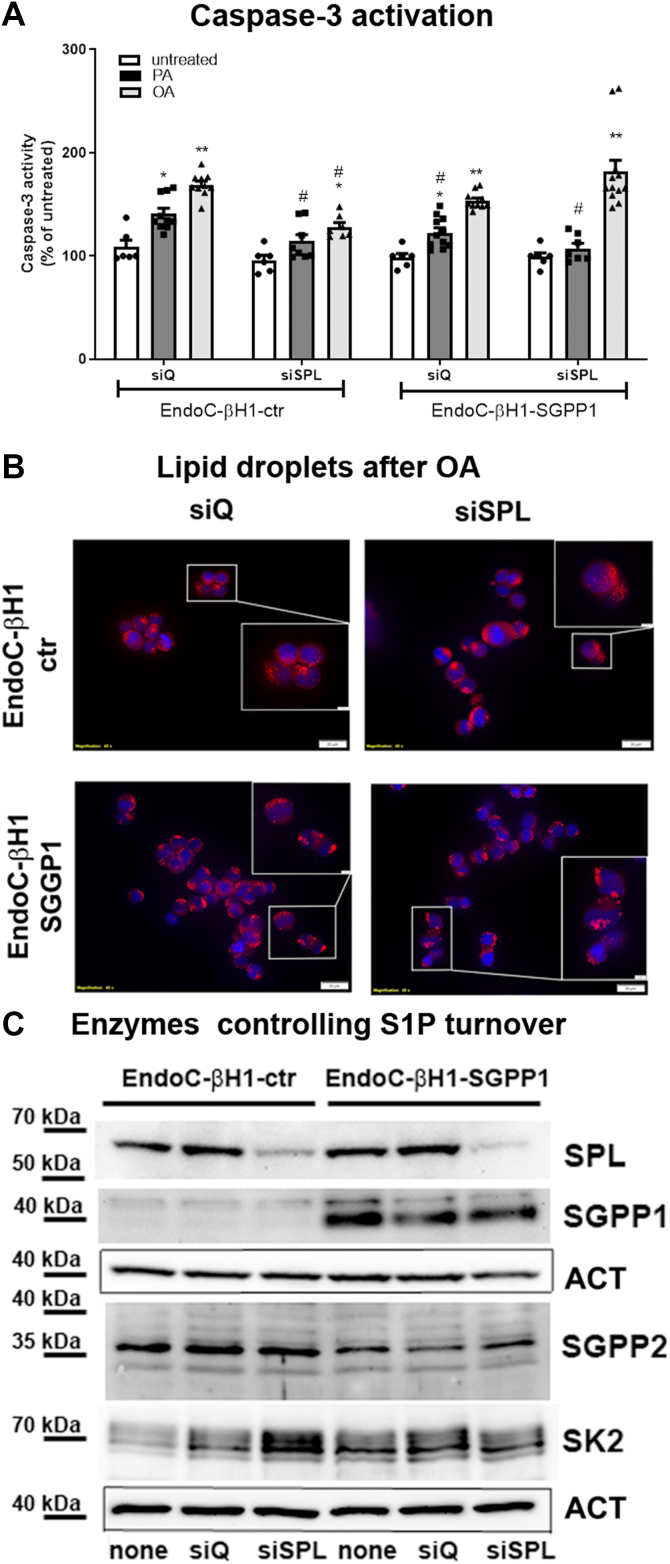
Effects of S1P turnover and free fatty acids on caspase-3/7 activation and lipid droplet formation in human EndoC-βH1 beta-cells. EndoC-βH1 beta-cells were incubated in the absence or presence of PA or OA (500 μM of each) for 24 h. Shown are (A) caspase-3/7 activation measured by Caspase-3/7-Glo assay (MEANS ± SEM from n = 6–10), (B) lipid droplet formation visualized by OilRed staining (representative pictures from n = 3), (C) representative Western blots for the detection of SPL, SGPP1, SGPP2, and SK2 from 3–4 independent experiments. ANOVA followed by Bonferroni, ∗*P* < 0.05, ∗∗*P* < 0.01, versus untreated, #*P* < 0.05, versus INS1E-ctr cells treated in the same way. Bars: 20 μm, inset bars: 2 μm.

In the last step of our study, we verified our findings from rat INS1E cells in the well-characterized human EndoC-βH1 beta-cells, which share a very similar glucose responsiveness and sensitivity to FFA with human islets (7, 28). Interestingly, the expression of SPL, but not of SGPP1, is significantly higher in EndoC-βH1 beta-cells as compared to INS1E cells (Fig. 9). For this reason, we modified the expression of SPL (siRNA-mediated suppression) and of SGPP1 (lentiviral-mediated overexpression) and analyzed the sensitivity to FFA (Fig. 9C). In line with former studies (5, 6, 7, 8), we observed significantly induced caspase-3/7 activation in response to PA as well as to OA in control EndoC-βH1 beta-cells, which was counteracted by SPL suppression (Fig. 9A). Interestingly, SGPP1 overexpression resulted in a significant reduction of PA-mediated caspase-3/7 activation but did not provide any protection against OA (Fig. 9A). Knockdown of SPL in SGPP1-overexpressing EndoC-βH1 beta-cells shut down the PA-related induction of apoptosis completely; however, did not prevent OA-mediated toxicity (Fig. 9A). Indeed, OA seemed to be slightly more toxic after SPL suppression than in EndoC-βH1-SGPP1 beta-cells with an abundant SPL expression.

Next, we analyzed the OA-mediated LD formation in EndoC-βH1 beta-cells with a genetically modified expression of SPL and/or SGPP1 (Fig. 9B). In line with earlier studies, we observed an increased LD formation in response to OA in control EndoC-βH1 beta-cells (Fig. 9B), which was enhanced after SPL suppression (Fig. 9B). In EndoC-βH1-SGPP1 cells, the number and size of LD was higher than in control cells (Fig. 9B). In EndoC-βH1-SGPP1 beta-cells with a reduced SPL expression, we observed numerous and particularly big LD, which tended to form clusters that filled the cytoplasm (Fig. 9B). Thus, the results obtained in human beta-cells confirm the distinct role of SPL versus SGPP1 in LD formation, which was observed in rat beta-cells. The lack of a protective effect after exposure to OA in EndoC-βH1-SGPP1 beta-cells with SPL knockdown correlated with the presence of unusual big and numerous LDs. This phenomenon of building enormously big hubs of LDs in EndoC-βH1 beta-cells under a parallel SPL suppression and SGPP1 overexpression might be related to an abundant expression of SCD1, an enzyme which has been recently shown to control LD formation in beta-cells (7, 40).

## Discussion

Disturbances of lipid metabolism and storage capacity have been suggested to contribute to lipotoxic beta-cell failure, but the underlying molecular mechanisms are still unclear. Interestingly, our study shows that the well-described effects of FFA (2, 5, 8, 9, 10, 11) are differentially affected by the stimulation of S1P turnover; yet in an opposite way: with proapoptotic effects of SPL and protection provided by SGPP1 overexpression. Apparently, the metabolic fate of S1P and the subcellular compartment in which S1P is metabolized determine whether a FFA exerts a toxic versus protective effect on beta-cells.

To uncover the mechanisms underlying these differential effects of SPL versus SGPP1, we analyzed in detail molecular pathways typically involved in lipotoxic beta-cell death. Our former study suggested that SPL overexpression might accelerate overall oxidative stress (8). We now further characterized the effects of two different paths of S1P metabolism on the generation of H_2_O_2_ in various cellular organelles. Our results demonstrate that overexpression of SPL but not of SGPP1 leads to an elevated FFA-induced H_2_O_2_ formation not only in peroxisomes, as typical H_2_O_2_ sources under lipotoxic stress in beta-cells (52, 63), but also in the cytosol and in mitochondria. Consistently, a higher expression of CuZnSOD (Tang, unpublished) and of MnSOD superoxide dismutases responsible for a higher capacity of H_2_O_2_ generation in the cytosol and in mitochondria, respectively, was identified. The increased SOD expression could additionally enhance the well-known imbalance of H_2_O_2_ formation and detoxification in beta-cells (48). In addition, the induction of mitochondrial H_2_O_2_ in PA- and—to a lesser extent—in OA-treated INS1E-SPL cells coincided with a significantly higher expression of CerS6, increased generation of Cer 14:0, Cer 16:0, and Cer 20:0, and mitochondrial ceramide accumulation as compared to INS1E-ctr cells. Former studies have demonstrated ceramide-induced oxidative stress in mitochondria and induction of apoptosis in other cell types (55, 64). Moreover, as reported for other cell types (65), elevation of H_2_O_2_ and of ceramide generation in FFA-treated INS1E-SPL cells correlated with profound mitochondrial fragmentation and hence with a drop of ATP content. Accordingly, CerS6-derived ceramides have been shown recently to interact with the mitochondrial fission factor to boost mitochondrial fragmentation in obesity (66) and to induce mitochondrial dysfunction and apoptosis in beta-cells (19, 27, 29). The vicious cycle leading to mitochondrial failure in FFA-treated INS1E-SPL cells was potentiated by a significantly lower expression of Cert1 paralleled by an increased expression of neutral sphingomyelinase and of SGPP2, thus impeding on the one hand ceramide transport to the Golgi and on the other hand providing more sphingosine, the substrate for efficient ceramide formation. An increased expression of neutral sphingomyelinase has been shown before to lead to ceramide generation in ER and mitochondria in INS1 beta-cells via an iPLA(2)beta-dependent pathway (67). Additionally, the observed deleterious effects of SPL overexpression on mitochondrial integrity could be also related to the reaction product of SPL, 2-hexadecenal, which has been shown to induce mitochondrial damage in other cell models (68). However, in line with the abundant expression of Aldh3a2 in INS1E-SPL cells, we were unable to detect hexadecenal by mass-spec, an observation which suggests its rapid metabolism and argues against its involvement in the observed higher toxicity of FFA in these cells. In contrast to INS1E-SPL cells, the mitochondrial network remained intact in INS1E-SGPP1 cells exposed to FFA, with a rich network of elongated mitochondria with a higher activity of mitochondrial fusion mechanisms. Moreover, unlike in INS1E-ctr and SPL overexpressing cells, the expression of Lonp1 was not stimulated upon FFA incubation, strengthening the notion that mitochondria of INS1E-SGPP1 cells were protected from FFA-mediated damage. Consistently, mitochondrial ceramide levels were significantly lower in these cells in which FFA treatment failed to stimulate CerS6 expression, while not affecting Cert1 expression. To the best of our knowledge, this is the first report visualizing mitochondrial ceramide distribution in beta-cells exposed to FFA and demonstrating the effects of S1P turnover pathways on mitochondrial integrity and ATP content in beta-cells.

In beta-cells, ceramide overload has also been associated with FFA-induced ER and oxidative stress (2, 19, 27, 28, 29, 30, 69). In the present study, we demonstrated that these deleterious events strongly depended on the irreversible degradation of S1P, but not on S1P as a starting point of the recycling pathway catalyzed by SGPP1. The SGPP1-mediated protective effects went along with a considerable inhibition especially of the rate-limiting step of the de novo sphingolipid biosynthesis. Moreover, the expression of CerS5, which produces proapoptotic C16 ceramide, was significantly lower in INS1E-SGPP1 cells as compared to INS1E-ctr and SPL-overexpressing cells. On the other hand, CerS2, which generates beta-cell protective ceramides (C-length >24) (70), was increased by FFA in INS1E-SGPP1 cells in a similar manner as in INS1E-ctr cells. Quantitive measurements revealed, however, that the main difference between FFA-treated cell clones was in the content of short-chain ceramides that are well known for their proapoptotic effects. Importantly, OA diminished the formation of such ceramides in INS1E-ctr cells in contrast to INS1E-SPL cells. Although many different ceramide species could be detected at low levels in INS1E-SGPP1 cells, the most abundant ceramide species in these cells was Cer 24:0 and Cer 24:1. This was in line with an abundant expression of Ormdl3 and lower amounts of SPTlc. Unlikely as in INS1E-SPL cells, where Cert1 protein expression was significantly lower than INS1E-ctr cells, in INS1E-SGPP1 cells, no reduction was observed and the basal expression of Cert1 indeed tended to be higher than the other cells studied. Altogether, this data suggest a shift of ceramide generation from proapoptotic short chain to long chain ceramide species and a dramatic transfer of these ceramides from the ER to the Golgi and to the plasma membrane or other cellular structures/organelles (e.g., lipid droplets), thereby preventing ceramide-mediated oxidative stress induction and damage of ER and mitochondria in INS1E-SGPP1 cells.

Former studies have shown that LDs can act as storage for different ceramide species and derivatives thereof (37). Accordingly, the number and size of LDs were both significantly enhanced in INS1E-SGPP1 cells as compared to control cells. In contrast, SPL overexpression blunted lipid storage capacity as shown by a reduced LD number and size. Accordingly, we observed a high number of LDs after suppression of SPL in OA-treated human EndoC-βH1 beta-cells. This effect was further potentiated by SGPP1 overexpression and accompanied by the formation of particularly large LD hubs as discussed above. The underlying mechanism of these changes could involve LD biogenesis and/or lipophagy. Intriguingly, the expression of seipin, an ER protein necessary for LD biogenesis, was found to be significantly increased only by OA in INS1E-SGPP1 and in control cells suggesting that additional factors are required for the observed changes of LDs in beta-cells under the studied conditions. One might be the composition of cellular lipids, which has been recently reported to play an important role for the expression and recruitment of specific Plin proteins to LD mantle in INS1E cells with an altered SCD1 expression and activity (40). SCD1-dependent disturbances in LD enrichment were shown to impact beta-cell susceptibility to PA (40). In contrast to INSE-SPL cells that were characterized by a significantly lower expression of Plin2 and Plin5, a significantly higher expression of these two LD proteins was detected in INS1E-SGPP1 cells. Plin5 was shown to protect against oxidative stress and mitochondrial damage in HepG2 cells (71) and to facilitate islet FA mobilization (61). An additional mechanism that could participate in a higher LD biogenesis in INS1E-SGPP1 cells could rely on the inhibited Dgat2 expression in response to FFA. Indeed, in kidney organoids, DGAT1 inhibition reduced LD number, while DGAT2 inhibition increased LD content via APOL1 and was associated with a drop of cytotoxicity (72). LD number can be also regulated by lipophagy, an autophagic pathway ending up by lysosomal degradation of LDs (73). The released neutral lipids are available for energy production and other reactions, which might lead to utilization of FA for the generation of bioactive lipids. A properly controlled lipophagy is believed to participate in the maintenance of cellular metabolism, while protecting from lipotoxicity (38). Therefore, INS1E-SGPP1 cells that were characterized by a blunted lipophagy could be more efficiently protected against lipotoxicity, but in turn obtain less fuel for ATP production. This was in contrast to a hyperactivated lipophagy, as observed in INS1E-SPL cells, which could result in undesired oversupply of FA for further metabolism and β-oxidation contributing to the induction of oxidative stress under FFA exposure. The hyperactive lipophagy in INS1E-SPL cells could be supported by a higher content of phosphatidylethanolamine, generated from phosphoethanolamine, a reaction product of SPL (44). Note that PE is recruiting and anchoring LC3 to autophagosomal membranes, an essential step for their formation and processing.

Intriguingly, the distinct effects of SPL and SGPP1 were largely related to oxidative stress but independent of FFA-mediated lipid peroxidation. PA, but not OA, induced lipid peroxidation in INS1E-ctr cells, that was significantly blunted by overexpression of both, SPL and SGPP1. It has been recently reported that H_2_O_2_ generated in peroxisomes, ER, and mitochondria during metabolic processes of FFA, and redox reactions is required for the membrane lipid peroxidation in INS1E beta-cells (46). In our study, SGPP1 overexpression inhibited FFA-induced H_2_O_2_ generation in the cytoplasm, peroxisomes, and mitochondria. This obviously constitutes a protective mechanism to prevent PA-induced lipid peroxidation. On the other hand, in INS1E-SPL cells, a considerable upregulation of H_2_O_2_ generation in various cellular compartments was also accompanied by a significant reduction of lipid peroxidation. A possible explanation of this paradoxical effect could be the fact that SPL represents a branching point of sphingolipid and glycerophospholipid metabolism, and changes of its activity were shown to largely affect membrane lipid composition (34). Thus, SPL overexpression might cause a change of the PUFA/MUFA ratio in membrane lipids in favor of MUFAs, known to prevent lipid peroxidation, as recently reported in OA-treated INS1E cells (46). Additionally, the lack of lipid peroxidation in SPL overexpressing cells emphasizes an essential role of proapoptotic ceramide generation particularly at the mitochondria/ER contact sides and its accumulation due to the lack of a proper lipid storage capacity for lipotoxicity in beta-cells.

Further investigations are needed to uncover the mechanisms involved in the observed changes regarding the expression of various sphingolipid metabolic enzymes as well as of mitochondrial and LD proteins. These changes may be related to the reported epigenetic regulation by S1P (74, 75), particularly in the case of INS1E-SPL cells, which display a considerably reduced S1P content. Although S1P concentration was also significantly reduced in INS1E-SGPP1 cells, the effect was less pronounced due to a parallel upregulation of SK2 and downregulation of SGPP2. Therefore, one would not expect a considerable effect on epigenetic regulation, especially in the case of genes subjected to more complex transcriptional regulatory mechanisms. However, changes of the lipidome as a result of SPL or SGPP1 overexpression might also affect the regulation of gene transcription via altered lipid signaling pathways.

**Fig. 10:**
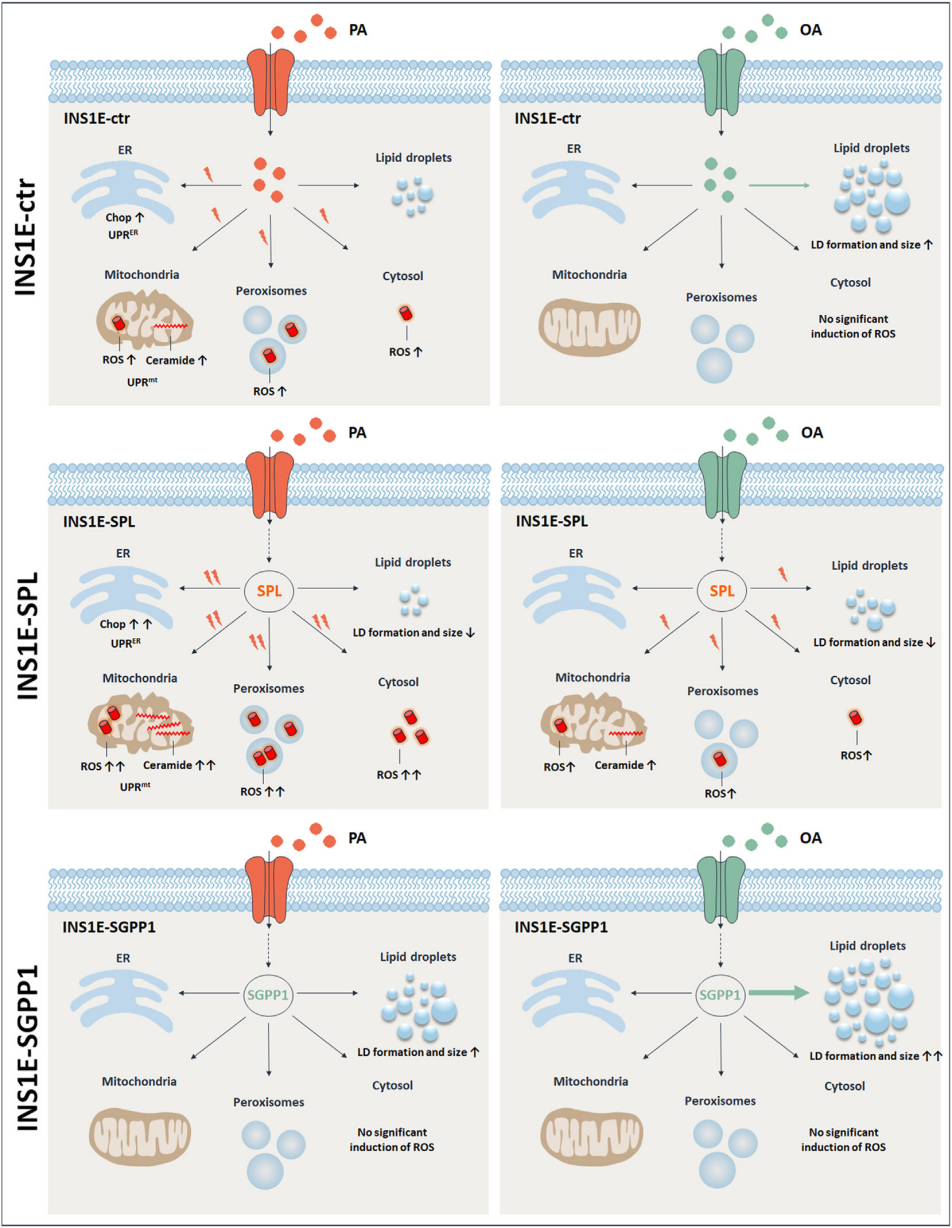
Schematic summary of the differential effects of SPL versus SGPP1 on FFA-mediated toxicity in pancreatic beta-cells. In INS1E-ctr cells, PA induces ER stress, mild mitochondrial stress related to the accumulation of proapoptotic ceramides in mitochondria and oxidative stress (H_2_O_2_ formation mainly in cytoplasm and peroxisomes), while OA is not toxic and stimulates lipid droplets formation. The enhanced S1P cleavage capacity by SPL overexpression accelerates toxic effects of PA and sensitized to OA via potentiation of oxidative and ER stress responses as well as reduction of lipid storage capacity accompanied by a massive accumulation of pro-apoptotic ceramides in mitochondria. SGPP1 overexpression protects against lipotoxicity via upregulation of lipid storage (LD particularly of a bigger size) and decrease in ceramide formation. For details with regard to human beta cells, look in Discussion. ER, endoplasmic reticulum, LD, lipid droplet, ROS, reactive oxygen species, UPR^ER^, ER unfolded protein response, UPR^mt^, mitochondrial unfolded protein response.

Finally, by validating our findings in human beta-cells, we confirmed the concept of distinct effects of SPL versus SGPP1 on FFA toxicity and LD formation. Thus, our study clearly shows that enzymes involved in S1P turnover are crucial for lipotoxic stress in beta-cells (summarized in Fig. 10). Our study also demonstrates that the sensitivity of beta-cells to FFA is not solely regulated by the intracellular S1P concentration per se but also by the metabolic pathway to which S1P is subjected and the subcellular compartments affected. Further studies are needed to investigate how the observed effects of intracellular S1P turnover in beta-cells might influence their sensitivity to FFA in a complex islet environment, with additional inputs of S1P and/or other bioactive metabolites produced and secreted from other islet cell types as well as exocrine tissue or islet vascular and innervation systems.

Overall, the results of the present study open new perspectives regarding the contribution of S1P turnover in beta-cells affected in T2DM patients. Hence, therapeutic strategies aiming on a specific and tight regulation of intracellular S1P turnover may turn out as an attractive tool for future pharmacological protection of beta-cell function in T2DM.

## Data availability

All relevant data for this study are included in this manuscript. The data which were not shown are available upon request from the corresponding author.

## Supplemental data

This article contains supplemental data.

## Conflict of interest

The authors declare that they have no conflicts of interests with the contents of this article.
