# Supplementary material for: The fate of intracellular S1P regulates lipid droplet turnover and lipotoxicity in pancreatic beta-cells
Source: J Lipid Res. 2024 Jun 29;65(8):100587. doi: 10.1016/j.jlr.2024.100587 (PMC11345310; doi:10.1016/j.jlr.2024.100587)
Supplement: supplemental Table S1 [file mmc1.docx]

**Supplementary Table S1. Primers used in real-time RT-PCR analysis.**

| Gene | FW | REV |
| --- | --- | --- |
| *Human SPL* | ACGGCCTGGTGGCATTA | CTGACAATTGGGGATTCCC |
| *Human SGPP1* | CCCGTGGTCAAGTTGGAGGTCT | ATAAGAGGGTACTGCCAGCGGC |
| *Rat SPTlc1* | TTGAAGAATGGCAGCCAGAG | CCGAGGAAATTAAAGGAGGC |
| *Rat SPTlc2* | GAAAAGTGCCACCATGCAAC | ACAGATGGGTCGATTCCAGT |
| *Rat 3Kdsr* | GCTCTGCAAATGGAGGTGAAGCC | TCTCTGAGATAAGCCGGGTCTCCAG |
| *Rat CerS2* | GCTTGCTTTCTACTGGTCCC | CTGCTCGGACATAATTGGCA |
| *Rat CerS5* | GCTGGCAGCAGGTCTCTTCT | CCCTTCAGCCTTTTCTCATC |
| *Rat CerS6* | CGGCATTATGTTCCTGCACCACC | CAGAGCATCGGCTGAGTCGTGAAG |
| *Rat Degs1* | AGACTTGGACTGGAAGTGGC | GGTTCCACATGGCCTTGTG |
| *Rat Dgat2* | CGGAGGCCACCGAAGTTAGCAA | AGGGCAGATGCCTCCAGACATCA |
| *Rat CD acid* | CAAGTGCCACCGTGGACAGA | GGTGCCTTGTGAGCCAATAA |
| *Rat CD neutral* | ACTCTGGCCCAGCAGGATT | GGACTTTGCCCGGTTTAAGA |
| *Rat SpM acid* | TCTTTGAGGACGATGTGGTG | GCACTGATGGCAAAGAGATG |
| *Rat SpM neutral* | GGCTGCTGGTGCTCCATCTA | TGGATGAACTGGGCCAGTT |
| *Rat Sk2* | CAAGCCCTACACATACAGCG | GCCACGTGGGTAGGTGTAGA |
| *Rat Sgpp2* | TTCTCCCATTCACCCACTG | TCACAACGGGAGGAGAGGAG |
| *Rat Seipin* | TGCTTCGCTCTGCTCTTTCCCTG | GGGGATTCTGGCAGCTCAAGCTCTA |
| *Rat Ormdl3* | AGCATCCCCTTTGTGAGCGTCC | TCCGGAGTCTCGAAGGGTGTCC |
| *Rat Aldh3a2* | CTCCCAACAGCGAGTCCAAG | ATCACAGCTGATCCTTGACGA |
| *Rat β-Actin* | GAACACGGCATTGTAACCAACTGG | GGCCACACGCAGCTCATTGTA |
