# Supplementary material for: The fate of intracellular S1P regulates lipid droplet turnover and lipotoxicity in pancreatic beta-cells
Source: J Lipid Res. 2024 Jun 29;65(8):100587. doi: 10.1016/j.jlr.2024.100587 (PMC11345310; doi:10.1016/j.jlr.2024.100587)
Supplement: supplemental Table S2 [file mmc2.docx]

**Supplementary Table S2. Antibodies used in Western blot and immunofluorescence experiments**

| **Antibody** | **Product data** | | **Company** |
| --- | --- | --- | --- |
| **SPL** | Rabbit polyclonal IgG, H-300, Cat # sc-67368  1:50 (IF) 1:500 (WB) | | Santa Cruz Biotechnology, Heidelberg, Germany |
| **SGPP1** | Rabbit polyclonal IgG, Cat # PA5-43467  1:100 (IF) 1:500 (WB) | | Thermo Fisher Scientific, Bremen, Germany |
| **SphK2** | Rabbit polyclonal IgG, Cat # 17096-1-AP  1:500 (WB) | | Proteintech, Planegg-Martinsried,  Germany |
| **SGPP2** | Rabbit polyclonal IgG, Cat # PA5-42767  1:500 (WB) | | Thermo Fisher Scientific, Bremen, Germany |
| **ALDH3A2** | Rabbit polyclonal IgG, Cat # 15090-1-AP  1:1000 (WB) | | Proteintech, Planegg-Martinsried,  Germany |
| **CERT1 (COL4A3BP)** | Rabbit polyclonal IgG, Cat # 15191-1-AP  1:500 (WB) | | Thermo Fisher Scientific, Bremen, Germany |
| **ORMDL3** | Rabbit polyclonal, Cat # ABIN2844388  1:500 (WB) | | Antibodies-online GmbH, Aachen,  Germany |
| **Cleaved Caspase3 (Asp175)** | Rabbit polyclonal, Cat # 9661  1:500 (WB) | | Cell Signalling Technology, Leiden, The Netherlands |
| **CHOP** | Rabbit monoclonal IgG, Cat # 5554  1:750 (WB) | | Cell Signalling Technology, Leiden, The Netherlands |
| **MnSOD** | Goat polyclonal IgG, G-20, Cat # sc-18504  1:500 (WB) | | Santa Cruz Biotechnology, Heidelberg, Germany |
| **LONP1** | Rabbit polyclonal IgG, Cat # PA5-51692, RRID: AB_2643424 1:750 (WB) | | Thermo Fisher Scientific, Bremen, Germany |
| **Phb2** | Mouse monoclonal IgG, A-2, Cat # sc-133094  1:500 (WB) | | Santa Cruz Biotechnology, Heidelberg, Germany |
| **Ceramide (MID 15B5)** | Mouse monoclonal IgM, Cat # ALX-804-196-T050  1:50 (IF) | | Biozol, Eching, Germany |
| **Plin 2** | Goat polyclonal IgG, C-20, Cat # sc-32450  1:500 (WB) | | Santa Cruz Biotechnology, Heidelberg, Germany |
| **Plin 3** | Mouse monoclonal IgG, E-5 Cat # sc-393461  1:1000 (WB) | | Santa Cruz Biotechnology, Heidelberg, Germany |
| **Plin 5** | Guinea pig polyclonal, Cat # GP31  1:500 (WB) | | PROGEN Biotechnik, Heidelberg, Germany |
| **β-Actin** | Mouse monoclonal IgG, C4, Cat # sc-47778  1:750 (WB) | | Santa Cruz Biotechnology, Heidelberg, Germany |
| **For SPL green** | **Secondary Alexa Fluor 488** | Donkey Anti-Rabbit IgG (H+L), Cat # 711-545-152  1:200 (IF) | Dianova, Hamburg, Germany |
| **For SGPP1 red** | **Secondary Alexa Fluor 647** | Donkey Anti-Rabbit IgG (H+L), Cat # 711-605-152  1:200 (IF) | Dianova, Hamburg, Germany |
| **For Ceramide green** | **Secondary Alexa Fluor 488** | Donkey Anti-Mouse IgG (H+L), Cat # 715-545-150  1:200 (IF) | Dianova, Hamburg, Germany |
| **For Plin5 green** | **Secondary Fluorescein (FITC)** | Donkey Anti-Guinea Pig IgG (H+L), Cat # 706-095-148  1:200 (IF) | Dianova, Hamburg, Germany |
